# In Vitro Synergistic Photodynamic, Photothermal, Chemodynamic, and Starvation Therapy Performance of Chlorin e6 Immobilized, Polydopamine-Coated Hollow, Porous Ceria-Based, Hypoxia-Tolerant Nanozymes Carrying a Cascade System

**DOI:** 10.1021/acsabm.3c01181

**Published:** 2024-02-21

**Authors:** Çağıl Zeynep Süngü Akdogan, Esin Akbay Çetin, Mehmet Ali Onur, Selis Önel, Ali Tuncel

**Affiliations:** † Bioengineering Division, Hacettepe University, Ankara 06800, Turkey; ‡ Graduate School of Science and Engineering, Hacettepe University, Ankara 06800, Turkey; § Department of Biology, Hacettepe University, Ankara 06800, Turkey; ∥ Chemical Engineering Department, Hacettepe University, Ankara 06800, Turkey

**Keywords:** cerium oxide, nanozyme, combinatorial therapy, multimodal therapy, hypoxia

## Abstract

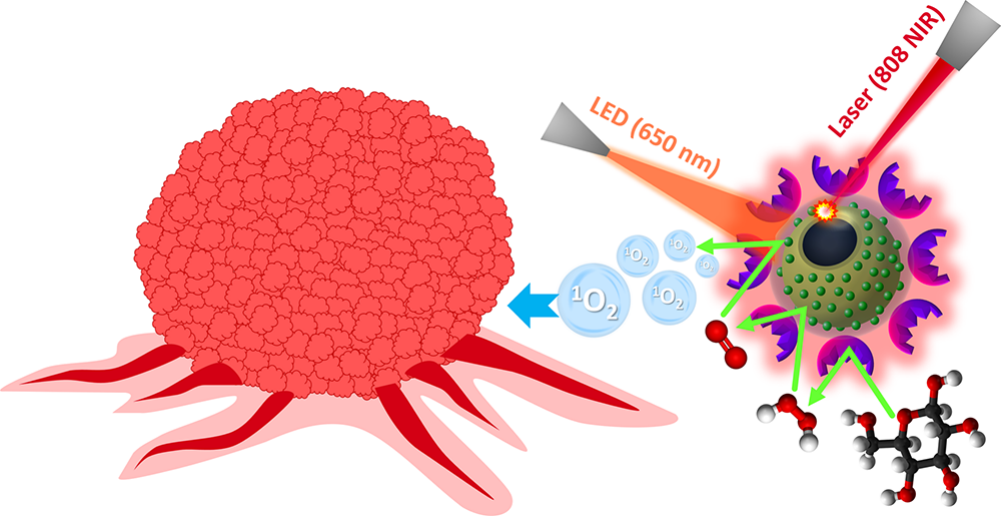

A synergistic therapy agent (STA) with photothermal, photodynamic, chemodynamic, and starvation therapy (PTT, PDT, CDT, and ST) functions was developed. Hollow, mesoporous, and nearly uniform CeO_2_ nanoparticles (H-CeO_2_ NPs) were synthesized using a staged shape templating sol–gel protocol. Chlorin e6 (Ce6) was adsorbed onto H-CeO_2_ NPs, and a thin polydopamine (PDA) layer was formed on Ce6-adsorbed H-CeO_2_ NPs. Glucose oxidase (GOx) was bound onto PDA-coated Ce6-adsorbed H-CeO_2_ NPs to obtain the targeted STA (H-CeO_2_@Ce6@PDA@GOx NPs). A reversible photothermal conversion behavior with the temperature elevations up to 34 °C was observed by NIR laser irradiation at 808 nm. A cascade enzyme system based on immobilized GOx and intrinsic catalase-like activity of H-CeO_2_ NPs was rendered on STA for enhancing the effectiveness of PDT by elevation of ROS generation and alleviation of hypoxia in a tumor microenvironment. Glucose-mediated generation of highly toxic hydroxyl radicals (^·^OH) was evaluated for CDT. The effectiveness of PDT on glioblastoma T98G cells was markedly enhanced by O_2_ generation started by the decomposition of glucose. A similar increase in cell death was also observed when ST and CDT functions were enhanced by photothermal action. The viability of T98G cells decreased to 10.6% by in vitro synergistic action including ST, CDT, PDT, and PTT without using any antitumor agent.

## 1 Introduction

In recent years, cerium oxide nanostructures (CeO_2_ NSs) have been widely evaluated as efficient tools in synergistic therapy applications.^1,2^ Photosensitizer-loaded ceria nanoparticles (CeO_2_ NPs) functionalized with polyethylenimine and polyethylene glycol were used in imaging-guided synchronous photochemotherapy.^3^ Nanoceria was evaluated for targeted photodynamic therapy (PDT) of drug-resistant breast cancer.^4^ Multimodal synergistic therapy applications were performed using various CeO_2_-based nanocomposites such as Pt@CeO_2_ core–shell NPs coated with MnO_2_, MnO_2_ nanoflowers carrying CeO_2_ QDs, CeO_2–*x*_@HA theranostic, Cu_2_O-coordinated g-C_3_N_4_ on CeO_2_ NPs, and MnO_2_-loaded CeO_2_ nanozyme.^5−9^ GOx-linked mesoporous SiO_2_/upconversion nanoparticle (UCNPs) composites and a metal organic framework decorated with CeO_2_ NPs were also used for combined photothermal/starvation therapy (PTT/ST) and PDT of tumors, respectively.^10,11^ SiO_*x*_/CeO_2_/VO_*x*_ composite nanozymes and Au@Pt nanospheres decorated with CeO_2_ NPs were evaluated for photothermal-catalytic therapy of tumors.^12,13^ Synergistic therapies including chemodynamic therapy (CDT) were applied using CeO_2_/CuO nanocomposite on UCNPs, NIR-triggered CeO_2_-based Janus nanomotors, MnO_2_ NPs decorated with CeO_2_, and dendritic mesoporous SiO_2_ NPs decorated with CeO_2_ and coated with a folic acid-linked polydopamine (PDA) layer.^14−17^ A pH-sensitive CeO_2_-ZIF-8 nanocomposite coated with PDA was also proposed for synergistic therapy of rheumatoid arthritis.^18^

Hypoxia in the tumor microenvironment seriously restricts the efficiency of PDT which is a strongly oxygen (O_2_)-dependent process.^19−22^ Nanozymes with catalase (CAT)-like activity have been attracted significant attention for alleviating hypoxia in the tumor microenvironment.^23^ A cascade enzyme system constructed by combining the activity of immobilized GOx with the CAT-like activity of various nanozymes is also an effective tool evaluated for hypoxia relief in synergistic therapy applications.^24−28^ On the other hand, GOx is mainly used for blocking glucose uptake by the tumor cells via starvation therapy (ST), which results in the inhibition of synthesis of heat shock proteins in cells.^29−32^

NIR responsiveness allowing the generation of heat in the tumor microenvironment makes PDA-based nanocarriers as one of the most widely preferred agents in synergistic therapy applications including PTT.^33−38^ In this study, PDA was evaluated as one of the main components of the targeted synergistic therapy agent (STA) constructed based on H-CeO_2_ NPs. As documented above, numerous studies have been performed using CeO_2_-based STAs toward application of combinatorial therapies with different configurations.^3−19^ Studies on the synthesis of CeO_2_-based STAs to apply synergistic therapies by alleviating hypoxia were also performed. For instance, HA/ICG-loaded CeO_2_ NPs were evaluated for PTT/PDT by alleviating hypoxia with MCF-7 cells.^39^ Pd1.7 Bi@CeO_2_ NSs loaded with ICG were used for PTT/PDT and CDT using 4T1 cells with hypoxia relief.^40^ A Ce6 and BSA-loaded CeO_2_/UCNP composite was synthesized for PDT with hypoxia relief on ID8 cells.^41^ GOx and Ce6-loaded CeO_2_ NPs were also tried for combined PDT/ST by ameliorating hypoxic conditions in HeLa cell culture.^42^

CeO_2_ nanostructures with a unique redox property based on the cycling between Ce(III) and Ce(IV) oxidation states exhibit CAT-like, peroxidase (POD)-like, and Fenton-like activities.^10,15,17,40,43,44^ CeO_2_ NPs have attracted significant attention in combinatorial therapy applications as nanoplatforms with Fenton-like activity which are capable of producing toxic ROS for tumor cells and as nanozymes with CAT-like activity, which react with H_2_O_2_ for producing O_2_ for hypoxia relief in the tumor microenvironment.^40^ There are several restrictions of CeO_2_ NPs that may reduce the effectiveness of synergistic therapy.^43^ The limited surface area of CeO2 NPs may lead to low adsorption capacities for some drugs and functional small biomolecules could be obtained with nonporous CeO_2_ NPs having a.^43,45^ Covalent linking of these agents onto CeO_2_ NPs may result in structural changes reducing their pharmacological activities.^43^ The limited surface area of nonporous CeO_2_ NPs might also hinder their enzyme-mimetic activity.

Recently, hollow CeO_2_ NPs with more effective enzyme-mimetic properties were developed as promising nanoplatforms for synergistic therapy applications.^43,44,46,47^ Low density, high surface area, large pore volume, and excellent therapeutic agent loading capacity are the major superiorities of hollow CeO_2_ NPs.^43,48−50^ Large voids are suitable containers for small functional guest nanostructures and various biological macromolecules such as enzymes that amplify the overall therapeutic performance with additional therapeutic functions or cascade reaction system.^44^ Hollow CeO_2_ NPs have a mesoporous shell with a low diffusion resistance facilitating either the transport of biological molecules or the interaction of functional molecules linked to STA with the biological agents in the tumor microenvironment.^43^ The mesoporous shell can protect a natural enzyme such as GOx which is immobilized on hollow CeO_2_ NPs and enhance its stability.^44^ The large surface area provided by the mesoporous shell facilitates the immobilization of a significant amount of GOx onto the hollow CeO_2_ particles. A nanoplatform with high CAT-like activity, glucose-mediated toxic ^·^OH radical generation ability, and enhanced morphological properties such as hollow CeO_2_ nanoparticles is an efficient tool for multifunctional synergistic therapy applications. Compared to nanoporous supports like metal–organic frameworks hollow CeO_2_ NPs exhibit superior acid stability, which is a crucial advantage considering the mildly acidic character of the tumor microenvironment.^51^

Hollow CeO_2_ NPs were evaluated in a limited number of studies on synergistic tumor therapy applications due to their potential advantages originating from large surface area and high volume for drug loading.^43,44,46,47^ In this context, doxorubicin-loaded hollow CeO_2_ NPs coated with PDA and ammonium bicarbonate and hollow CeO_2_ nanozymes that target lysosomes were used for synergistic therapy of tumors.^43,46^ Mesoporous, hollow CeO_2_ microspheres were also evaluated as an enzyme nanoreactor for catalytic antibacterial therapy.^44^ Dual chemotherapy (CT) and photothermal therapy (PTT) for colorectal cancer was applied using Ru@CeO_2_ yolk shell nanozymes as the synergistic agents.^47^ Hollow CeO_2_ nanoparticle-based therapeutic nanoplatforms have been invesitgated for their potential in synergistic therapy applications including PDT combined with hypoxia relief, CDT combined with hypoxia relief, combination of PTT, and chemotherapy (CT) combination and even triple combinations of PTT, CT, and CDT.^43,44,46,47^

Motivated by these promising applications, we developed a novel method for synthesizing mesoporous, hollow CeO_2_ nanoparticles with tunable mean size (H-CeO_2_ NPs). Chlorin e6 (Ce6), a common photosensitizer approved by the FDA was adsorbed onto H-CeO_2_ NPs and a thin NIR-responsive PDA shell was generated on the Ce6-adsorbed CeO_2_ NPs. The CAT-like activity of H-CeO_2_ NPs enabled the construction of a cascade enzyme/nanozyme system on a targeted STA through GOx immobilization. This led to the development of a new H-CeO_2_-based nanoplatform (H-CeO_2_@Ce6@PDA@GOx NPs) exhibiting in vitro synergistic therapy functions, including PTT, PDT, CDT, and ST while simultaneously alleviating hypoxia. The effectiveness of the H-CeO_2_@Ce6@PDA@GOx nanocomposite for in vitro synergistic therapy was demonstrated using T98G glioblastoma cells. This multifunctional nanoplatform allowed ST by depleting glucose in the T98G culture medium. The CAT-like activity of these NPs led to the decomposition of the H_2_O_2_, which was generated by the oxidation of glucose, into O_2_ for hypoxia relief within the tumor microenvironment. H_2_O_2_ was further consumed by the cytotoxic ^·^OH radicals produced via the Fenton-like activity of H-CeO_2_ NPs that mediate for simultaneous application of CDT with ST.^10,15,17,40,44^ An appreciable improvement in the effectiveness of PDT was observed by O_2_ generation initiated by decomposition of glucose via the cascade nanozyme/enzyme system. The effectiveness of ST and CDT was markedly enhanced by the temperature elevation induced by photothermal conversion ability of H-CeO_2_@Ce6@PDA@GOx NPs.

## 2 Experimental Section

### 2.1 Synthesis of H-CeO_2_ NPs

The porous H-CeO_2_ nanozyme was synthesized using a new staged shape templating sol–gel method based on a previous protocol proposed for the synthesis of compact-porous CeO_2_ microspheres.^52,53^ The synthesis method is given in Section S2 of the Supporting Information.

### 2.2 Preparation of Ce6-Loaded H-CeO_2_ NPs

Ce6 was loaded onto H-CeO_2_ NPs via physical adsorption.^54^ Typically, H-CeO_2_ NPs (5.0 mg/mL) were mixed with Ce6-methanol solution (1.0 mg/mL, 1.0 mL). The adsorption of Ce6 onto H-CeO_2_ NPs was carried out at room temperature and in the dark under magnetic stirring at 300 rpm for 30 min. H-CeO_2_@Ce6 NPs were washed with Tris buffer (10 mM, pH 8.5) using a centrifugation/decantation protocol. Ce6 adsorption onto H-CeO_2_ NPs was determined by a spectrophotometric protocol.^55^ For this purpose, the adsorption medium was centrifuged at 6000 rpm for 10 min, and the absorbance of the supernatant was measured at 400 nm in a UV–vis spectrophotometer (Thermo Scientific, Genesys 150, USA). Ce6 adsorption onto H-CeO_2_ NPs, shown by *Q*_Ce6_, was calculated based on the following equation:
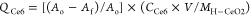
1where *A*_o_ and *A*_f_ are the absorbance values of the initial Ce6-methanol solution and the supernatant obtained after the adsorption, respectively. *C*_Ce6_ (mg/mL) is the initial concentration of Ce6 in the methanol solution. *V* (mL) is the volume of the adsorption medium and M_H-CeO2_ (g) is the mass of H-CeO_2_ NPs.

### 2.3 Preparation of PDA-Coated H-CeO_2_@Ce6 NPs

H-CeO_2_@Ce6 NPs (5 mg) were dispersed in Tris buffer (2.5 mL, 10 mM, pH: 8.5) by ultrasonication for 2 min. Dopamine hydrochloride (DA-HCl, 1.0 mg) was added rapidly, and the resulting dispersion was magnetically stirred at 400 rpm, for 90 min at room temperature.^56^ PDA shell-coated H-CeO_2_@Ce6 NPs (H-CeO_2_@Ce6@PDA NPs) were extensively washed with DI water by centrifugation/decantation and redispersed in phosphate buffer (25 mM, pH 7.0).

### 2.4 Immobilization of Glucose Oxidase (GOx) on H-CeO_2_@Ce6@PDA NPs

GOx (1 mg) was physically adsorbed onto H-CeO_2_@Ce6@PDA NPs (5.0 mg) in phosphate buffer (1.0 mL, 25 mM, pH 7.0).^57^ The dispersion was rotated at 50 rpm for 30 min, in the dark. The resulting H-CeO_2_@Ce6@PDA@GOx NPs were washed with DI water by a successive centrifugation/decantation protocol.

### 2.5 Catalase-like and Peroxidase-like Activity of H-CeO_2_@PDA NPs

CAT-like activity and POD-like activity of H-CeO_2_ NPs and H-CeO_2_@PDA NPs were determined using the protocols given in Section S4 of the Supporting Information.^2,58^

### 2.6 GOx Activity of H-CeO_2_@PDA@GOx NPs

The activity GOx immobilized on H-CeO_2_@PDA NPs was determined by following the protocol given in Section S5 of the Supporting Information.^59,60^ GOx binding onto H-CeO_2_@Ce6@PDA NPs was determined using a Bradford assay (Sigma-Aldrich, MO, USA, B6916).

### 2.7 Monitoring of Hydroxyl Radical Generation by Fluorescence Spectroscopy

The generation of ^·^OH radicals by H-CeO_2_@PDA@GOx NPs in the presence of glucose was shown by a fluorescent assay using 2-hydroxyterephthalic acid (2-HTPA) as the probe.^17,44^ For this purpose, a control sample was prepared by dissolving 0.5 mM terephthalic acid (TPA) and 5.0 mM H_2_O_2_ in DI water (12 mL). Two samples were prepared by dispersing H-CeO_2_@PDA@GOx NPs at different concentrations in an aqueous solution (12 mL) containing TPA (0.5 mM) and glucose (5.0 mM). The concentrations of H-CeO_2_@PDA@GOx NPs in these samples were set to 1 and 2 mg/mL. Each medium was magnetically stirred in the dark for 1 h at 350 rpm, at 37 °C for the formation of a highly fluorescent product, 2-HTPA via the reaction between TPA and ^·^OH radicals generated in the medium containing glucose.^17,44^ H-CeO_2_@PDA@GOx NPs were separated from the aqueous part by centrifuging at 6000 rpm for 10 min. The fluorescence spectrum of each supernatant was recorded using a fluorescence spectrophotometer (Shimadzu, RF-5301PC, Japan) with the excitation at a wavelength of 315 nm.

### 2.8 Photothermal Properties of H-CeO_2_@Ce6@PDA@GOx NPs

Photothermal response of H-CeO_2_@Ce6@PDA@GOx NPs was measured by using a NIR laser (808 nm, Kenar Mühendislik, Turkey). An aqueous dispersion of H-CeO_2_@Ce6@PDA@GOx NPs (200 μL) was exposed to the NIR laser for 5 min at a power density of 3.2 W cm^–2^, following the protocol given in Section S6 of the Supporting Information.

### 2.9 Photodynamic Response of H-CeO_2_@Ce6@PDA@GOx NPs

Photodynamic response of H-CeO_2_@Ce6@PDA@GOx NPs was determined by monitoring the production of singlet oxygen (^1^O_2_) radicals using 1,3-diphenylisobenzofuran (DPBF) as a chemical probe based on the protocol given in Section S7 of the Supporting Information.^55^

### 2.10 Intracellular ROS Generation by H-CeO_2_@Ce6@PDA@GOx NPs

For the detection of intracellular reactive oxygen species (ROS), T98G cells were seeded into a 98-well plate with a cell density of 2 × 10^4^ cells/well and cultured in a 5% (v/v) CO_2_ atmosphere at 37 °C for 24 h. H-CeO_2_@Ce6@PDA@GOx NPs were introduced to the cells at different concentrations. The cells were consecutively irradiated with 808 nm NIR laser and 650 nm red LED for 5 and 7 min, respectively. The medium was replaced with dichloro-dihydrofluorescein diacetate (DCF-DA) solution (10 μM), and the cells were incubated for 2 h. After washing the cells with PBS, the images showing live/dead cells were obtained using fluorescence microscopy.^61^

### 2.11 Combined Photothermal, Photodynamic, Chemodynamic, and ST Performance of H-CeO_2_@Ce6@PDA@GOx NPs

Synergistic therapy experiments using H-CeO_2_@PDA, H-CeO_2_@Ce6@PDA, and H-CeO_2_@Ce6@PDA@GOx NPs were performed based on the protocol given in Section 8 of the Supporting Information.^62^ The combinatorial PDT/PTT/CDT/ST-induced cell death was investigated with dual cell staining using the acridine orange/propidium iodide (AO/PI) system.^63,64^ In vitro cytotoxicity of H-CeO_2_ and H-CeO_2_@Ce6@PDA@GOx NPs was also investigated using the L929 subcutaneous connective tissue cell line. The detailed protocols are given in Section 8 of the Supporting Information.

## 3 Results and Discussion

### 3.1 Characterization of STA (H-CeO_2_@Ce6@PDA@GOx NPs)

Uniform H-CeO_2_ NPs were obtained based on a staged shape templating sol–gel protocol using poly(GDM-*co*-MA) particles as the seed latex. To start the synthesis of H-CeO_2_ NPs, uniform, cross-linked poly(GDM-*co*-MA) particles 0.45 μm in size were obtained by a previously developed precipitation polymerization protocol based on the copolymerization of glycerol dimethacrylate (GDM) and methacrylic acid (MA) in toluene–acetonitrile mixture.^53^ This protocol allows the synthesis of uniform poly(GDM-*co*-MA) particles in the size range of ca. 0.1–1.5 μm by adjusting the feed concentration of comonomers and the volume ratio of toluene/acetonitrile.^53^ In the present study, uniform CeO_2_/poly(GDM-*co*-MA) composite particles were synthesized by the formation of a mesoporous CeO_2_ shell on poly(GDM-*co*-MA) core particles in an aqueous alkaline medium. In the last stage, mesoporous H-CeO_2_ NPs with an approximate shell thickness of 100 nm are obtained by the removal of polymer-based core from the composite particles via calcination at 550 °C. The developed protocol allows for the synthesis of almost uniform, mesoporous H-CeO_2_ NPs with a prescribed size based on the selection of poly(GMA-*co*-MA) seed particles in a size range of 0.1–1.5 μm.

**Figure 1:**
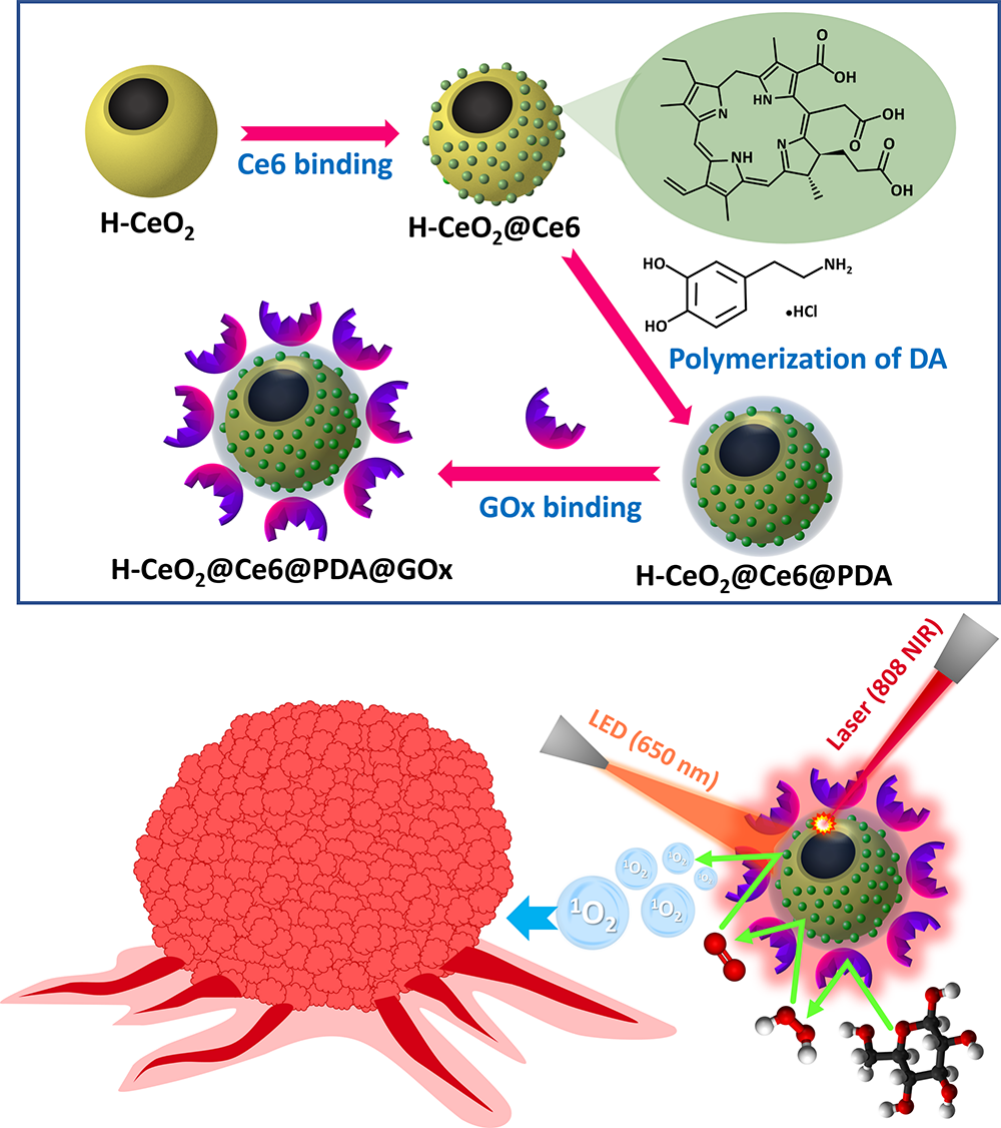
Chemical route followed for the synthesis of H-CeO_2_@Ce6@PDA@GOx NPs and the usage of STA for in vitro combinatorial therapy with TG98G cells.

**Figure 2:**
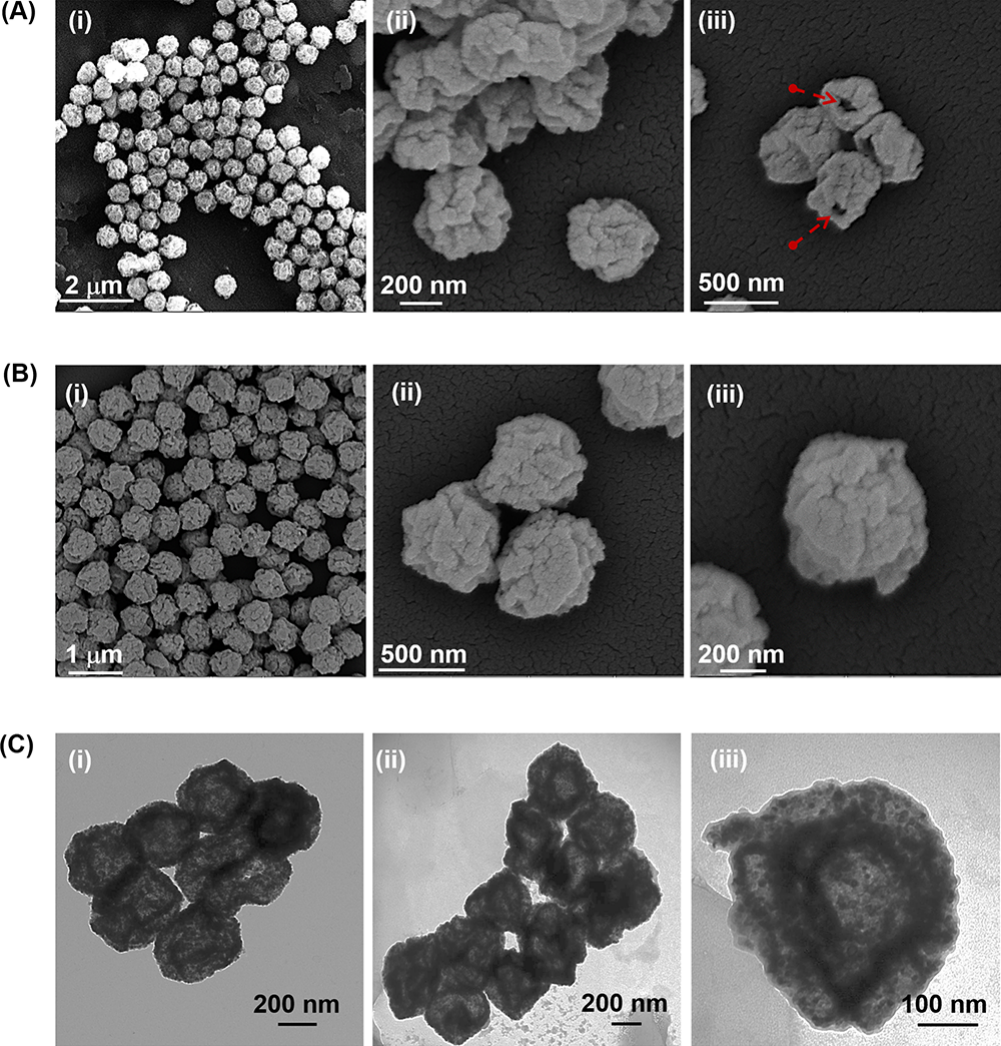
(A) SEM photographs of H-CeO_2_ and (B) H-CeO_2_@Ce6@PDA@GOx NPs. (C) TEM photographs of H-CeO_2_@Ce6@PDA@GOx NPs. Magnification: (A) (i) 25,000×, (ii) 140,000×, (iii) 100,000×, (B) (i) 35,000×, (ii) 115,000×, (iii) 154,000×, (C) magnification: (i) 50,000×, (ii) 50,000×, (iii) 150,000×. The red arrows in Figure 2A(iii) show the holes of H-CeO_2_ NPs.

**Figure 3:**
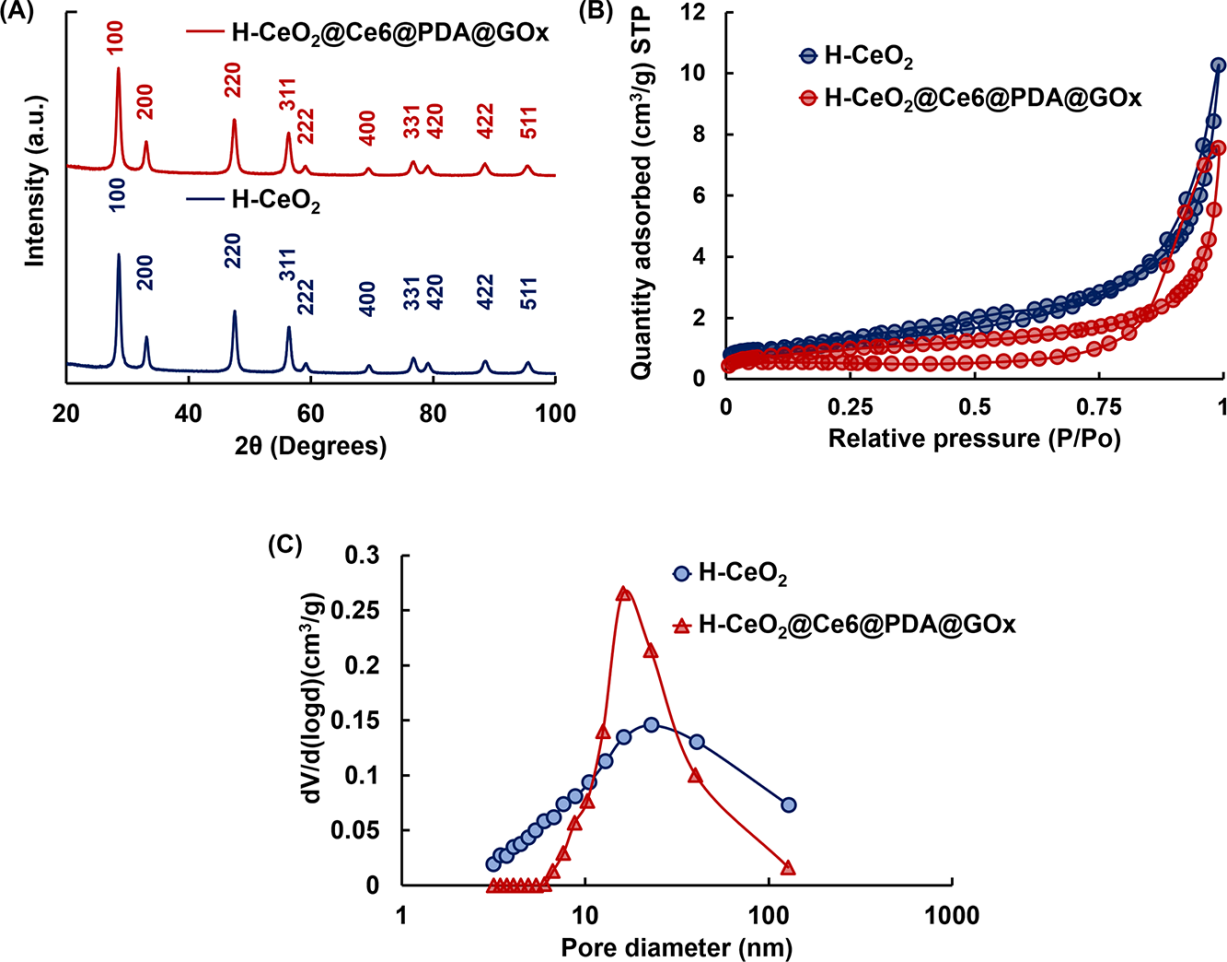
(A) XRD patterns, (B) nitrogen physisorption isotherms, and (C) pore size distribution curves of H-CeO_2_ and H-CeO_2_@Ce6@PDA@GOx NPs.

The chemical route followed for the synthesis of H-CeO_2_@Ce6@PDA@GOx NPs is given in Figure 1. The selected photosensitizer, Ce6 was physically adsorbed onto H-CeO_2_ NPs in methanolic solution. A thin PDA layer was formed on the Ce6-adsorbed H-CeO_2_ NPs by polymerization of dopamine in Tris buffer at pH 8.5. GOx was bound onto H-CeO_2_@Ce6@PDA NPs in phosphate buffer at pH 7. The obtained STA (H-CeO_2_@Ce6@PDA@GOx NPs) was evaluated for in vitro combinatorial therapy with PTT, PDT, CDT, and ST functions using T98G glioblastoma cells (Figure 1). Scanning electron microscope (SEM) photographs of H-CeO_2_ and H-CeO_2_@Ce6@PDA@GOx NPs are given in Figure 2. As seen in Figure 2A(i), H-CeO_2_ NPs were synthesized with considerably narrow size distribution almost uniform in size, using the developed staged shape templating sol–gel protocol. The mean size of H-CeO_2_ NPs was determined as 540 nm (Table S1 of the Supporting Information). No significant change is expected in both the mean size and uniformity of the H-CeO_2_ particles based on the functionalization protocol applied toward obtaining H-CeO_2_@Ce6@PDA@GOx NPs (Figure 2B). The mean size of H-CeO_2_@Ce6@PDA@GOx NPs was determined as 550 nm. The slight increase in the mean size is due to the formation of a PDA shell on H-CeO_2_ NPs. The hollow form of H-CeO_2_ NPs is clearly observed in Figure 2A(iii). Energy-dispersive X-ray spectroscopy (EDX) images of H-CeO_2_ and H-CeO_2_@Ce6@PDA@GOx NPs are given in Figure S1A,B, respectively. EDX analysis confirms the presence of Ce, O, and C atoms on H-CeO_2_ NPs as expected (Figure S1A). The observed C signal is attributed to the residual carbon from decomposition of the polymethacrylate template during calcination. The same atoms are observed in the EDX images of H-CeO_2_@Ce6@PDA@GOx NPs (Figure S1B). Additionally, N and S atoms originating from the PDA shell and bound GOx, respectively, are observed in the EDX images of H-CeO_2_@Ce6@PDA@GOx NPs. TEM photographs of H-CeO_2_@Ce6@PDA@GOx NPs demonstrating the hollow form are given in Figure 2C. In the X-ray diffraction (XRD) spectra of H-CeO_2_ and H-CeO_2_@Ce6@PDA@GOx NPs, the diffraction peaks at 2θ angles associated with the (111), (200), (220), (311), (222), (400), (331), (420), (422), (333), (440), (531), and (600) planes were assigned to the face-centered cubic fluorite phase of CeO_2_ (ICDD34–0394) (Figure 3A). The average crystallite size values for H-CeO_2_ and H-CeO_2_@Ce6@PDA@GOx NPs were calculated as 12.55 and 10.89 nm, respectively, consistent with previous reports on ceria nanoparticles.^65,66^ Notably, the functionalization protocol induce no significant change in the crystalline structure of H-CeO_2_ core as evidenced by the preserved fluorite phase in the XRD pattern of H-CeO_2_@Ce6@PDA@GOx NPs. The porous properties of H-CeO_2_ and H-CeO_2_@Ce6@PDA@GOx NPs are given in Table S1. As seen in Figure 3, HB-CeO_2_ and H-CeO_2_@Ce6@PDA@GOx NPs exhibited a Type IV adsorption isotherm corresponding to mesoporous solids, including monolayer and multilayer hysteresis and capillary condensation in desorption. H-CeO_2_ NPs had a broader pore size distribution located in the range of 3–130 nm with a mean pore size of 23.0 nm (Figure 3C and Table S1). The surface area of H-CeO_2_ NPs was higher than that of H-CeO_2_@Ce6@PDA@GOx NPs likely due to the higher volume fraction of small pores in the range of 3–9 nm (Table S1 and Figure 3C). Surface area and pore volume moderately decreased by the formation of PDA shell on H-CeO_2_ NPs (Table S1). This phenomenon did not affect the surface area and the pore volume of H-CeO_2_@Ce6@PDA@GOx NPs that remained still satisfactorily high. The formation of PDA shell on H-CeO_2_ NPs resulted in a narrower pore size distribution and a lower mean pore size compared to those obtained for H-CeO_2_ NPs (Figure 3C). Notably a large pore volume and high surface area are the superiorities that provide advantages in the potential loading of chemotherapeutic agents onto H-CeO_2_@Ce6@PDA@GOx NPs.

**Figure 4:**
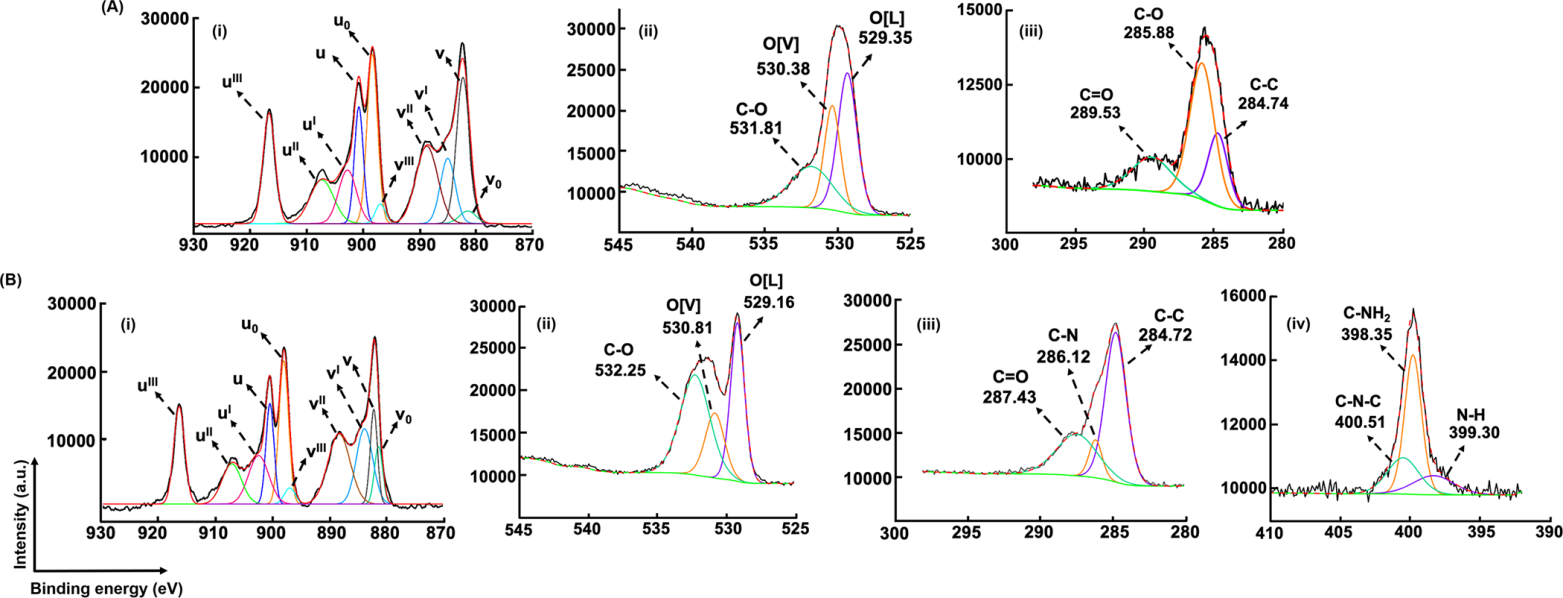
X-ray photoelectron spectroscopy of H-CeO_2_ NPs and H-CeO_2_@Ce6@PDA@GOx NPs. (A) Core level spectra for (i) Ce 3d scan, (ii) O 1s scan, and (iii) C 1s scan of H-CeO_2_ NPs. (B) Core level spectra for (i) Ce 3d scan, (ii) O 1s scan, (iii) C 1s scan, and (iv) N 1s scan of H-CeO_2_ NPs H-CeO_2_@Ce6@PDA@GOx NPs.

Survey X-ray photoelectron spectra (XPS) of H-CeO_2_ and H-CeO_2_@Ce6@PDA@GOx NPs are given in Figure S2 of the Supporting Information. In the survey XPS of H-CeO_2_ NPs, Ce 3d, O 1s, and C 1s peaks were observed at 883.08, 530.08, and 285.08 eV, respectively. For H-CeO_2_@Ce6@PDA@GOx NPs, Ce 3d, O 1s, and C 1s peaks are located at the binding energies of 883.08, 531.08, and 285.08 eV, respectively. On the other hand, N 1s peak was obtained at 400.08 eV in survey XPS of H-CeO_2_@Ce6@PDA@GOx NPs. The presence of N 1s peak should be evaluated as an indicator for the formation of PDA shell on H-CeO_2_ NPs. The core level spectra for Ce 3d scan for H-CeO_2_ and H-CeO_2_@Ce6@PDA@GOx NPs are given in Figure 4A(i),B(i), respectively. According to Burrough’s approach, the atomic fractions of Ce(III) and Ce(IV) are calculated as the ratio of respective peak for a certain component to the total area of 10 peaks obtained by deconvolution as given in eq 2.^2^
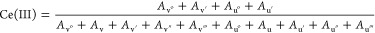
2

Based on this approach, six peaks (u, u″, u‴, v, v″, and v‴) are assigned to Ce(IV) and four peaks (u^o^, u′, v^o^, and v′) are associated with Ce(III) (Figure 4A(i),B(i)).^2,67^ The surface atomic concentrations of Ce(III) and Ce(IV) are given in Table S2. Notably, concentrations of Ce(III) and Ce(IV) are considerably lower for H-CeO_2_@Ce6@PDA@GOx NPs with respect to those of H-CeO_2_ NPs. The atomic composition calculated from C 1s peak is higher for H-CeO_2_@Ce6@PDA@GOx NPs. These two findings can be explained by the formation of PDA shell on H-CeO_2_ NPs. In the core level spectra for O 1s scan, the peaks that belong to lattice oxygen, surface adsorbed oxygen due to oxygen vacancy and −C–O bonds were obtained for both H-CeO_2_ and H-CeO_2_@Ce6@PDA@GOx NPs (Figure 4A(ii),B(ii)).^68,69^ Different from the core level spectra for C 1s scan of H-CeO_2_ NPs, a peak at 286.12 eV associated with the C–N bond, originating from the PDA shell was observed in the core level spectra for C 1s scan of H-CeO_2_@Ce6@PDA@GOx NPs (Figure 4A(iii),B(iii)). The peaks obtained at 389.30, 398.35, and 400.51 eV in the core level spectra for N 1s scan of H-CeO_2_@Ce6@PDA@GOx NPs are assigned to the −N–H, −C–NH_2_, and −C–N–C– groups, respectively (Figure 4B(iv)).^13,65,66^

### 3.2 Multifunctional Enzyme-Mimetic Behavior of STA

In order to determine the enzyme-mimetic activities of synthesized STA, the photosensitizer (i.e., Ce6) unloaded form (i.e., H-CeO_2_@PDA@GOx NPs) was used due to the release of Ce6 from H-CeO_2_@Ce6@PDA@GOx NPs during the determination of enzyme-mimetic activity by the colorimetric protocols.^70^ The presence of Ce6 in the aqueous medium results in an interference in the absorbance measurements performed for the determination of enzyme-mimetic activities by the colorimetric protocols.

**Figure 5:**
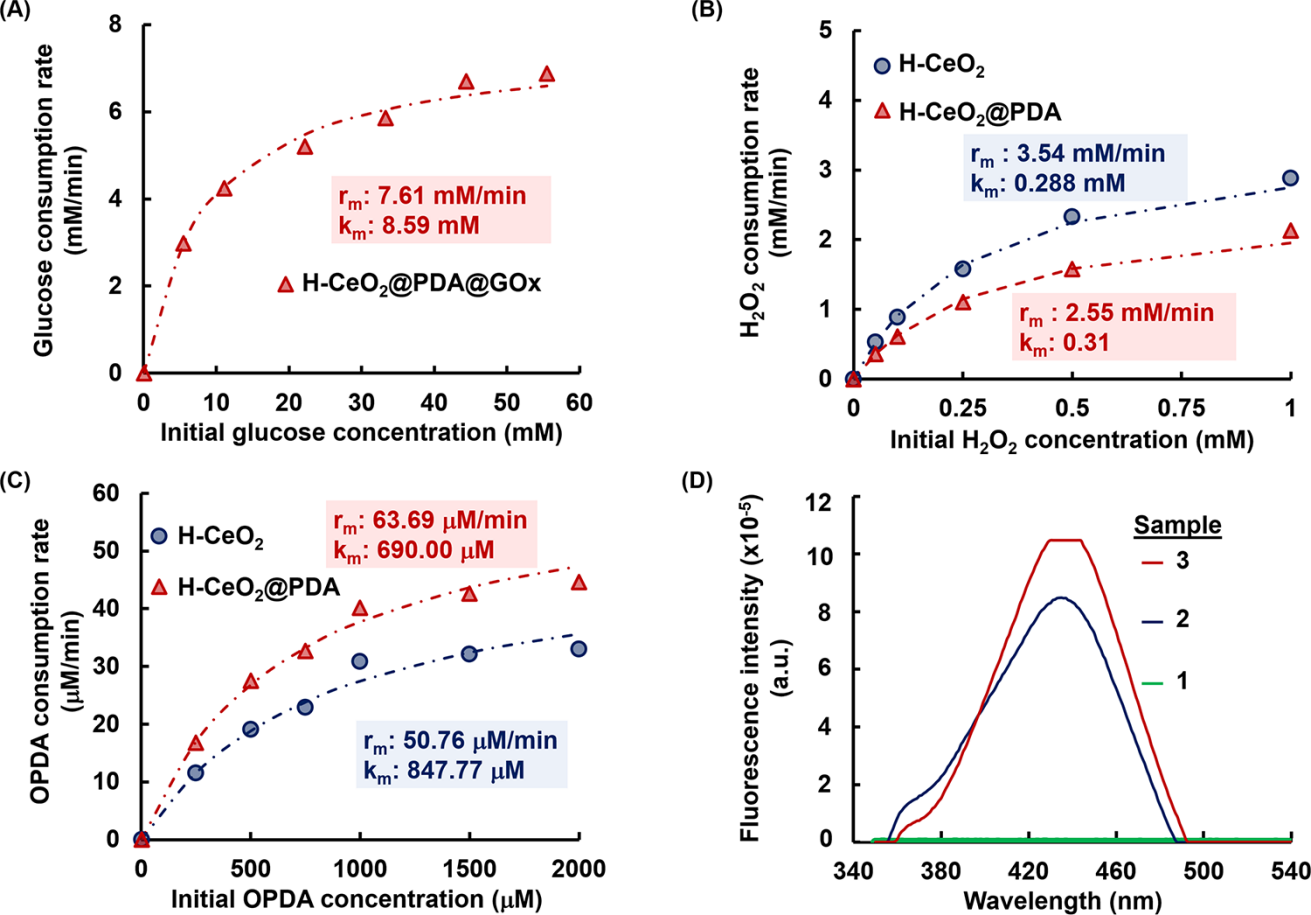
Plots showing GOx, catalase-like, and peroxidase-like activities and ^·^OH radical generation ability of H-CeO_2_-based STAs synthesized at different forms. (A) Michaelis–Menten plot for GOx activity of H-CeO_2_@PDA@GOx NPs. Nanozyme concentration: 2 mg/mL. Temperature: 37 °C. (B) Michaelis–Menten plot for catalase-like activity of H-CeO_2_ and H-CeO_2_@PDA NPs. Nanozyme concentration: 2 mg/mL. Temperature: 22 °C. (C) Michaelis–Menten plot for peroxidase-like activity of H-CeO_2_ and H-CeO_2_@PDA NPs. Nanozyme concentration: 2 mg/mL. Temperature: 22 °C. (D) Fluorescence spectra of TPA oxidation catalyzed by H-CeO_2_@PDA@GOx NPs. Legends: sample (1) control: 0.5 mM TPA + 5 mM H_2_O_2_, sample (2) 0.5 mM TPA + 1 mg/mL H-CeO_2_@PDA@GOx NPs + 5 mM glucose, sample (3) 0.5 mM TPA + 2 mg/mL H-CeO_2_@PDA@GOx NPs + 5 mM glucose. Conditions: magnetic stirring in dark at 37 °C for 1 h, excitation: 315 nm, emission: 435 nm.

GOx activity of H-CeO_2_@PDA@GOx NPs was determined at pH 7. A Lineweaver–Burk plot for GOx activity is given with a high correlation coefficient in Figure S3. The Michaelis–Menten plot and the values of *V*_max_ and *K*_m_ were determined based on the Lineweaver–Burk plot and are given in Figure 5A. A good consistency is observed between the experimental glucose consumption rates and the values calculated using the Michaelis–Menten model for H-CeO_2_@PDA@GOx NPs.

The equilibrium GOx binding onto H-CeO_2_@Ce6@PDA NPs was determined as 126 mg GOx/g H-CeO_2_@PDA@GOx NPs. High GOx loading can be attributed to large surface area and pore volume originating from the mesoporous character of H-CeO_2_@Ce6@PDA NPs. The activity of free GOx is given as 17.6 μmol β-D-glucose/mg GOx.min (Sigma-Aldrich, G6125). The maximum glucose consumption rate (*V*_max_) obtained for the GOx immobilized-H-CeO_2_@Ce6@PDA NPs was found as 7.61 mM glucose/min based on the Michaelis–Menten model (Figure 5A). This value corresponds to a glucose consumption rate of 22.8 μmol glucose/min for a reaction volume of 3 mL used in the activity runs performed with 12 mg of H-CeO_2_@Ce6@PDA@GOx NPs (Section S5 of the Supporting Information). The maximum glucose consumption rate that can be obtained with the amount of free enzyme, which is equivalent to the amount immobilized on 12 mg of H-CeO_2_@Ce6@PDA@GOx NPs is calculated as 26.6 μmol glucose/min based on the given catalog value. The comparison of glucose consumption rates calculated for free and immobilized GOx shows that 85.7% of the activity of free GOx is obtained with the immobilized enzyme when H-CeO_2_@Ce6@PDA NPs are used as the support. This value shows that GOx is successfully immobilized on H-CeO_2_@Ce6@PDA NPs by preserving the activity of free enzyme to a large extent.

On the other hand, H_2_O_2_ decomposition profiles obtained with H-CeO_2_, H-CeO_2_@PDA and H-CeO_2_@PDA@GOx NPs based on their CAT-like activity are exemplified in Figure S4A. H_2_O_2_ was decomposed completely due to the CAT-like activity of H-CeO_2_ and H-CeO_2_@PDA NPs in 60 min under the studied conditions. H-CeO_2_@PDA@GOx NPs exhibited a slightly lower H_2_O_2_ decomposition rate with respect to H-CeO_2_ and H-CeO_2_@PDA NPs. Either the formation of PDA shell or binding of GOx did not cause a marked decrease in the CAT-like activity of H-CeO_2_ NPs. The Lineweaver–Burk plots sketched for the determination of Michaelis–Menten model parameters for catalase-like activity of H-CeO_2_ and H-CeO_2_@PDA@GOx NPs are given in Figure S4B. Linear relations with satisfactorily high correlation coefficients are obtained between the inverse of H_2_O_2_ decomposition rate and the inverse of H_2_O_2_ initial concentration for both H-CeO_2_ and H-CeO_2_@PDA@GOx NPs. Michaelis–Menten plots for CAT-like activity of H-CeO_2_ and H-CeO_2_@PDA@GOx NPs are given in Figure 5B. As expected, lower *V*_max_ and higher *K*_m_ were found for H-CeO_2_@PDA@GOx NPs with respect to those obtained for H-CeO_2_ NPs (Figure 5B). A good consistency between experimentally determined and predicted substrate consumption rates for both H-CeO_2_ and H-CeO_2_@PDA@GOx NPs demonstrated that the relationship between substrate consumption rate and initial substrate concentration can be adequately described by the Michaelis–Menten model.

Variation of the POD-like activity of H-CeO_2_@PDA NPs with pH is shown in Figure S5A. The maximum POD-like activity was obtained at neutral pH. Lineweaver–Burk plots belonging to POD-like activity of H-CeO_2_ and H-CeO_2_@PDA NPs are given in Figure S5B. The Michaelis–Menten plot and *V*_max_ and *K*_m_ values for the POD-like activity of H-CeO_2_ and H-CeO_2_@PDA NPs are given in Figure 5C. Higher *V*_max_ and lower *K*_m_ were found for peroxidase-like activity of PDA-coated H-CeO_2_ NPs. As previously mentioned, PDA contains catechol/o-quinone couples and a redox mediator contributing to its individual catalytic activity.^71^ The individual enzyme activities including the CAT-like activity originated from H-CeO_2_ NPs and the activity of immobilized GOx on PDA-coated H-CeO_2_ NPs confirmed the presence of a cascade enzyme system on H-CeO_2_@PDA@GOx NPs.

In the present case, POD-like activity of H-CeO_2_@PDA NPs was determined by the formation of 2,3-diaminophenazine from *o*-phenylenediamine (OPDA) as the substrate in the presence of H_2_O_2_.^12,44^ This reaction involves the generation of ^·^OH radicals by the interaction of H-CeO_2_@PDA NPs with H_2_O_2_ based on their Fenton-like activity and the interaction of produced ^·^OH radicals with OPDA for the formation of 2,3-diaminophenazine.^12,44^ The formation of ^·^OH radicals by the Fenton-like activity of H-CeO_2_@PDA@GOx NPs was monitored by a fluorescent assay, using TPA as the precursor of the fluorescent probe.^17,44^ The fluorescence spectra of the control sample and the supernatants obtained from the aqueous dispersions containing TPA, glucose, and H-CeO_2_@PDA@GOx NPs are given in Figure 5D. In the presence of oxygen, glucose is converted to H_2_O_2_ and gluconic acid by means of GOx immobilized on H-CeO_2_@PDA NPs. The produced H_2_O_2_ is evaluated by H-CeO_2_@PDA@GOx NPs via their Fenton-like activity, for the generation of ^·^OH radicals which convert nonfluorescent TPA into a highly fluorescent compound, 2-HTPA (λ_em_ = 435 nm).^12,17,44^ No appreciable fluorescence intensity was detected in the wavelength range of 350–550 nm in the control sample (1) containing TPA and H_2_O_2_ (Figure 5D). Hence, no significant amount of 2-HTPA is formed in 1 h in the control sample including no H-CeO_2_@PDA@GOx NPs. However, apparent fluorescence intensities at 435 nm which is the characteristic emission wavelength for 2-HTPA are observed for TPA-glucose solutions containing H-CeO_2_@PDA@GOx NPs at two different concentrations [i.e., 1.0 mg/mL in sample (2) and 2.0 mg/mL in sample (3)]. Here, the fluorescence intensity also increased with the increasing concentration of H-CeO_2_@PDA@GOx NPs, which indicates the formation of additional 2-HTPA likely due to the generation of more ^·^OH in the presence of larger amount of H-CeO_2_@PDA@GOx NPs.^12,17,44^ Hence, both the POD-like activity and the fluorescence assay clearly demonstrate the ^·^OH radical generation ability of H-CeO_2_@PDA@GOx NPs in the presence of glucose (Figure 5C,D). The conversion of nontoxic glucose to the ^·^OH radical which is highly toxic for the tumor microenvironment demonstrates the CDT function of H-CeO_2_@PDA@GOx NPs originating from their Fenton-like activity.^12,17,44^ Note that highly toxic ^·^OH radical production ability and its evaluation for CDT have been demonstrated using various CeO_2_-based STAs against different tumor cells.^7,10−13,15,17,40,46^

### 3.3 Photothermal Conversion Behavior of STA

**Figure 6:**
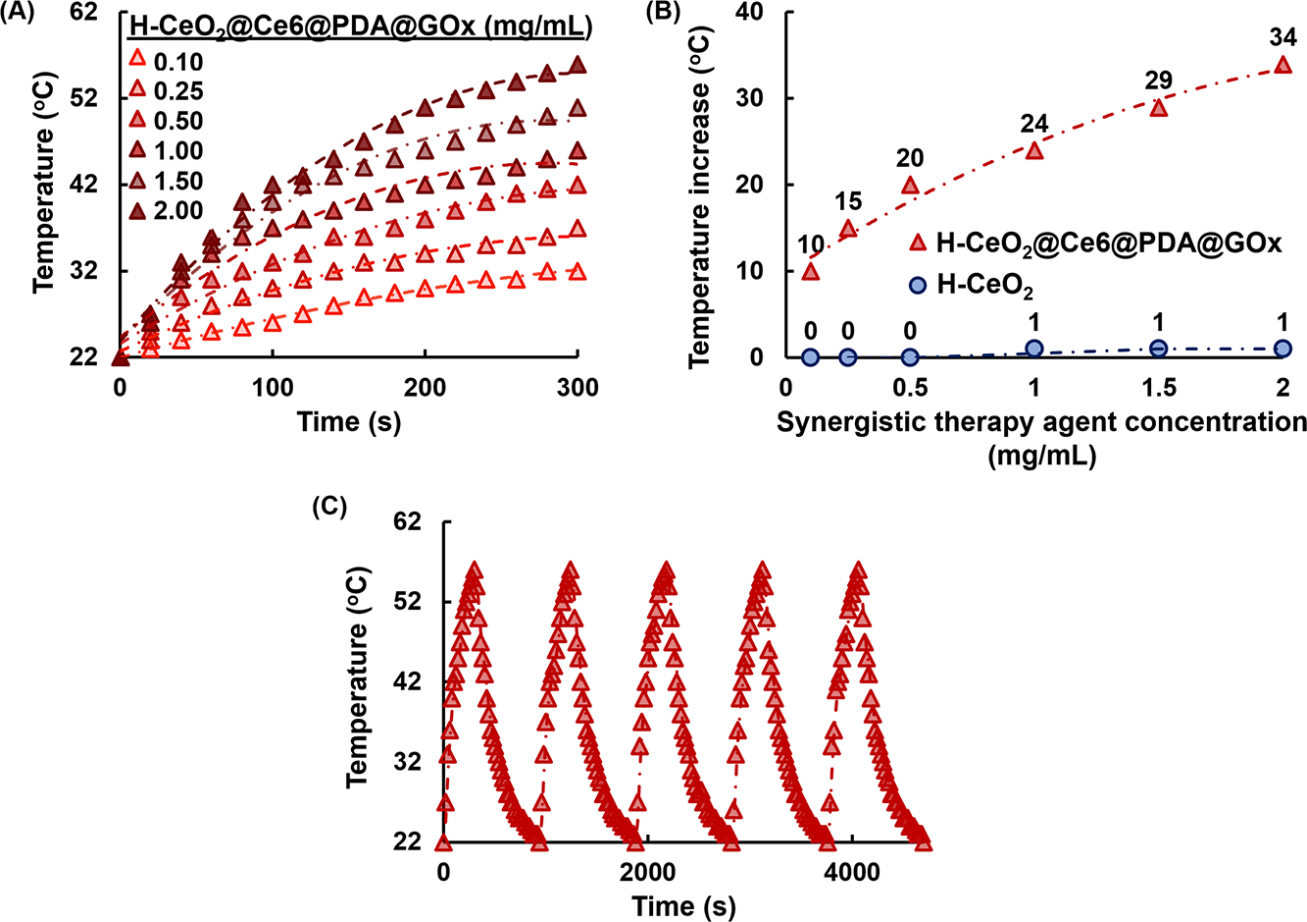
(A) Temperature elevation curves obtained with different concentrations of H-CeO_2_@Ce6@PDA@GOx NPs under NIR light (808 nm) irradiation: power density: 3.2 mW/cm^2^. (B) Variation of temperature elevation in 5 min with the concentration of H-CeO_2_ and H-CeO_2_@Ce6@PDA@GOx NPs. (C) Consecutive heating/cooling curves by turning on/off of NIR laser in the presence of H-CeO_2_@Ce6@PDA@GOx NPs at a concentration of 2 mg/mL.

Temperature elevation curves obtained with different concentrations of H-CeO_2_@Ce6@PDA@GOx NPs under NIR laser light (808 nm) irradiation are given in Figure 6. The temperature elevation was monitored for 5 min at each concentration. An appreciable photothermal conversion behavior was observed with H-CeO_2_@Ce6@PDA@GOx NPs exhibited under laser light irradiation, while no significant temperature increase was obtained with H-CeO_2_ NPs (Figure 6A,B). The photothermal conversion efficiency (η) was calculated as 42% using eq S1 with the time constant of 175.6 s found from the cooling curve obtained for H-CeO_2_@Ce6@PDA@GOx NPs at concentration of 2 mg/mL (Figure S6).^2,72,73^ This finding showed that PDA coating on H-CeO_2_ NPs provided a remarkable photothermal response to the synthesized STA. As expected, the total increase in temperature became higher with the increasing concentration of H-CeO_2_@Ce6@PDA@GOx NPs. The elevation in temperature reached 34 °C for the highest concentration of H-CeO_2_@Ce6@PDA@GOx NPs used in these runs (i.e., 2 mg/mL). The successive heating/cooling curves obtained by turning the NIR laser on and off in the presence of H-CeO_2_@Ce6@PDA@GOx NPs at a concentration of 2 mg/mL are presented in Figure 6C. No significant change occurred both in the temperature elevation and reduction curves with the increasing cycle number up to 5 runs. This behavior showed the stability of photothermal response of H-CeO_2_@Ce6@PDA@GOx NPs and also the usability of this nanomaterial as a new agent for PTT.

### 3.4 Photodynamic Response of STA

**Figure 7:**
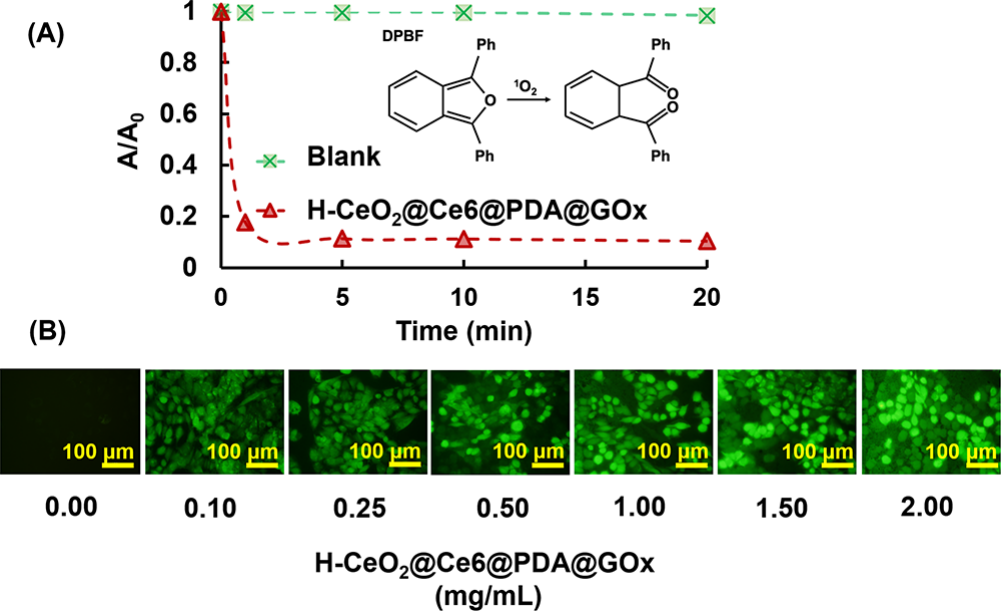
(A) Generation of ^1^O_2_ radicals in the presence of H-CeO_2_@Ce6@PDA@GOx NPs under LED light at 650 nm. H-CeO_2_@Ce6@PDA@GOx NPs concentration: 5 mg/mL, irradiation time: 20 min. Initial DPBF concentration: 0.03 mg/mL, volume: 3 mL. (B) Inverted fluorescence microscope images obtained with different concentrations of H-CeO_2_@Ce6@PDA@GOx NPs demonstrating the intracellular ROS formation under LED light irradiation at 650 nm. H-CeO_2_@Ce6@PDA@GOx NPs concentration: 0–2 mg/mL. T98G concentration: 2 × 10^4^ cells/well, irradiation time: 7 min (0.8 W). Scale bar: 100 μm.

Ce6 binding onto H-CeO_2_ NPs was determined as 70.3 mg Ce6/g H-CeO_2_ NPs. This value demonstrated that the selected photosensitizer was effectively adsorbed onto H-CeO_2_ NPs. The generation of singlet oxygen (^1^O_2_) radicals by exposing H-CeO_2_@Ce6@PDA@GOx NPs to visible light irradiation obtained from a red LED at 650 nm was followed by a spectrophotometric protocol, using DPBF as the fluorescent probe.^55^ The variation of DPBF concentration with the time in nonaqueous dispersion of H-CeO_2_@Ce6@PDA@GOx NPs exposed to visible light irradiation was determined by plotting the maximum absorbance of the supernatant obtained from the sample at 410 nm against time (Figure 7A). The sudden decrease of absorbance demonstrated that almost all of the DPBF was consumed rapidly by the ^1^O_2_ radicals generated, while no significant change was observed in the absorbance of the control medium. ^1^O_2_ radical production ability of H-CeO_2_@Ce6@PDA@GOx NPs under visible light irradiation at 650 nm was confirmed by this plot.

In order to have an idea about the effect of ^1^O_2_ radical production on T98G cells in the presence of H-CeO_2_@Ce6@PDA@GOx NPs exposed to visible light irradiation at 650 nm, intracellular ROS formation was followed using DCF-DA as the fluorescent probe.^61,74^ The inverted fluorescence microscope images obtained with different concentrations of H-CeO_2_@Ce6@PDA@GOx NPs under red LED light irradiation are given in Figure 7B. As seen here, the green fluorescence emission obtained by excitation at 485 nm increased with the increasing concentration of H-CeO_2_@Ce6@PDA@GOx NPs. In other words, an appreciable increase in the formation of intracellular ROS was obtained with the increasing concentration of H-CeO_2_@Ce6@PDA@GOx NPs exposed to visible light irradiation at 650 nm. Figure 7 clearly shows that H-CeO_2_@Ce6@PDA@GOx NPs is a potential PDT agent that can be used with visible light irradiation. The measured value of the total intracellular ROS is likely to include ^1^O_2_ radicals generated by exposure of H-CeO_2_@Ce6@PDA@GOx NPs to visible light irradiation at 650 nm and the ^·^OH radicals generated by H-CeO_2_@Ce6@PDA@GOx NPs using the glucose already found in the culture medium used for observing intracellular ROS formation.

### 3.5 In Vitro Combinatorial Therapy on T89G Cells Using STA

In the first set, the control runs were performed with T98G cells using only using LED, only using NIR laser, only using LED + NIR laser in the absence of STA, and also only using H-CeO_2_ NPs and H-CeO_2_@Ce6@PDA@GOx NPs at a concentration of 1 mg/mL, in the absence of a light source. The representative images of T98G cells stained with AO/PI after the control runs are given in Figure S7A. MTT results obtained in the control runs are given in Figure S7B. As seen in Figure S7A, no significant intracellular red fluorescence indicating the presence of dead cells was detected in the control runs. This finding was also supported by the MTT results given in Figure S7B. The percentage of viable cells were obtained as 97.23, 96.69, and 95.70% when red LED, NIR laser, and LED+NIR laser, respectively, were used as the light source in the absence of H-CeO_2_@Ce6@PDA@GOx NPs. The percentage of viable cells was 98.03% when H-CeO_2_@Ce6@PDA@GOx NPs were interacted with T98G cells in the absence of a light source (Figure S7B). This finding also indicated that only the consumption of glucose by GOx immobilized on H-CeO_2_@Ce6@PDA@GOx NPs and ^·^OH radicals generated by H-CeO_2_@Ce6@PDA@GOx NPs in an interaction period of 5 min were not so effective for killing tumor cells in the absence of a light source. H-CeO_2_ NPs were also included in the control runs in the absence of a light source. Satisfactorily high cell viabilities were observed for both types of NPs.

The in vitro cytotoxicity of H-CeO_2_ and H-CeO_2_@Ce6@PDA@GOx NPs was investigated using L929 subcutaneous connective tissue cell line as the healthy cells. After treatment with H-CeO_2_ and H-CeO_2_@Ce6@PDA@GOx NPs at different concentrations, the live/dead L929 cell images and MTT results demonstrating the viability of L929 cells are given in Figure S8A,B of the Supporting Information, respectively. No significant cell death was observed in the presence of H-CeO_2_ and H-CeO_2_@Ce6@PDA@GOx NPs in the concentration range of 0.1–2 mg/mL (Figure S8A). MTT results also provided L929 cell viabilities higher than 96% even if the concentration was increased up to 2 mg/mL for both NPs (Figure S8B).

T98G cells were interacted with H-CeO_2_@PDA NPs and H-CeO_2_@Ce6@PDA NPs using NIR laser at 808 nm and red LED at 650 nm, respectively. The representative live/dead cell images of T98G cells stained with AO/PI after treatment with H-CeO_2_@PDA NPs and H-CeO_2_@Ce6@PDA NPs using NIR laser and red LED, respectively, are given in Figure S9A. In these images, live and dead cells were observed with intracellular green and red fluorescence emissions, respectively. MTT results obtained in these runs are included in Figure S9B. The run performed by using H-CeO_2_@PDA NPs with NIR laser irradiation at 808 nm shows the effect of only PTT on T98G cells since the selected therapy agent does not contain Ce6 and immobilized GOx. In this case, the single factor which is responsible for cell death is the photothermal response of H-CeO_2_@PDA NPs induced by NIR laser irradiation. The run performed with H-CeO_2_@Ce6@PDA NPs using red LED at 650 nm gives the effect of only PDT on T98G cells since the ROS generated by the interaction of Ce6 with visible light is the single factor which is responsible for cell death. As shown in Figure S9A, the intracellular red fluorescence was not dominant in the representative live/dead cell images of T98G cells stained with AO/PI after treatment with H-CeO_2_@PDA NPs using NIR laser at 808 nm and H-CeO_2_@Ce6@PDA NPs using red LED at 650 nm. The viabilities of T98G cells in the runs showing the effects of only PTT (H-CeO_2_@PDA NPs using NIR laser at 808 nm) and only PDT (H-CeO_2_@Ce6@PDA NPs using red LED at 650 nm) were determined as 69.84 and 71.22%, respectively (Figure S9B). The live/dead cell images were supported by the MTT results. This behavior showed that limited cell deaths could be obtained when only PTT or only PDT was applied with H-CeO_2_ NP-based synergistic agents and also demonstrated the requirement of a combinatorial approach for obtaining satisfactory cell deaths with the intended STA.

**Figure 8:**
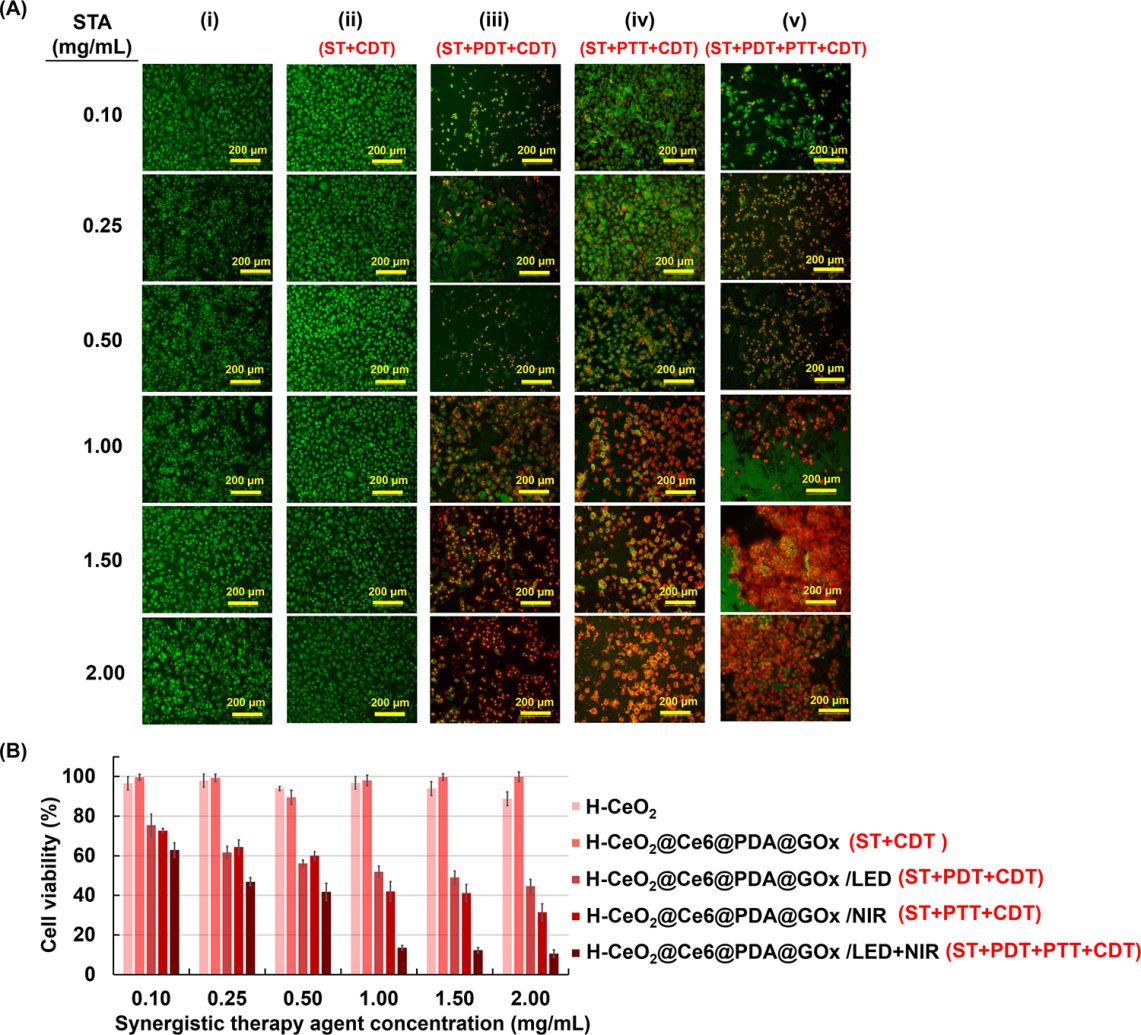
(A) Representative live/dead cell images of T98G cells stained with AO/PI after treatment with different concentrations of STAs. (i) H-CeO_2_ NPs, (ii) H-CeO_2_@Ce6@PDA@GOx NPs, (iii) H-CeO_2_@Ce6@PDA@GOx NPs/LED, (iv) H-CeO_2_@Ce6@PDA@GOx NPs/NIR laser, and (v) H-CeO_2_@Ce6@PDA@GOx NPs/LED + NIR laser. Scale bar: 200 μm. (B) MTT results demonstrating the viability of T98G cells after treatment under different conditions using STAs at different concentrations. T98G concentration: 2 × 10^4^ cells/well, LED irradiation time: 7 min (0.8 W), NIR irradiation time: 5 min (3.2 W cm^–2^).

For this purpose, T98G cells were interacted with H-CeO_2_@Ce6@PDA@GOx NPs at different concentrations using NIR laser and/or red LED as the light sources. The representative live/dead cell images of T98G cells stained with AO/PI after interaction are given in Figure 8A. As seen here, intracellular red cells fluorescence emissions showing the dead cells were dominant with H-CeO_2_@Ce6@PDA@GOx NP concentrations in the range of 1.0–2 mg/mL when NIR laser and red LED were used together. MTT results obtained by the interaction of T98G cells with H-CeO_2_@Ce6@PDA@GOx NPs at different concentrations using NIR laser and/or red LED are given in Figure 8B. The comparison of Figure 8A,B shows that the fluorescence microscopy images were supported by the MTT results. The cell viabilities obtained with different concentrations of H-CeO_2_@Ce6@PDA@GOx NPs in the absence of a light source were higher than 95% in most cases (Figure 8B). These results indicated that the starvation function of the STA based on the consumption of glucose via immobilized GOx was not so effective for cell death under the studied conditions. The glucose consumption rate which is not satisfactorily high for complete consumption of glucose in the presence of high initial glucose concentration (i.e., 4.5 mg/mL) and the limited treatment time (i.e., 5 min) should be likely the reasons preventing the increase in dead cells by the starvation function of STA. The insufficient glucose consumption rate observed in the absence of a light source indicates a low ^·^OH radical production rate which also reduces the effect of CDT. On the other hand, the cell viabilities observed in the absence of light sources can also be evaluated that no significant cytotoxicity for T98G cells, originated from H-CeO_2_@Ce6@PDA@GOx NPs, is detected in the concentration range of 1–2 mg/mL.

As known, the oxidation of glucose by means of GOx immobilized on STA results in the formation of gluconic acid and H_2_O_2_ in the tumor microenvironment. The formed H_2_O_2_ is then decomposed into O_2_ by catalase-like activity of STA. The generation of O_2_ should positively contribute to alleviation of hypoxia in a tumor microenvironment. More effective PDT should be obtained in the presence of hypoxia relief since an enhancement in the ROS generation rate is expected by increasing O_2_ concentration in the tumor microenvironment. Hence, the presence of GOx on STA should positively contribute to the synergistic therapy function by increasing the effectiveness of PDT. The main findings obtained in the synergistic therapy experiments performed with H-CeO_2_@Ce6@PDA@GOx NPs can be listed as follows:

(i) The viability of T98G cells was determined as 71.22% when H-CeO_2_@Ce6@PDA NPs with a concentration of 1 mg/mL were exposed to visible light irradiation by red LED at 650 nm for 7 min (Figure S8B). However, the cell viability decreased to 51.95% when H-CeO_2_@Ce6@PDA@GOx NPs was exposed to visible light irradiation under the same conditions (Figure 8B). Hence, higher concentration of dead cells in T98G cell-culturing medium in which O_2_ evolution was triggered by the cascade enzyme system on H-CeO_2_@Ce6@PDA@GOx NPs should be explained by the ROS generation with higher rate in the presence of higher O_2_ concentration.

(ii) A similar decrease in the viability of T98G cells was also observed when PTT performance of H-CeO_2_@PDA NPs was compared with that of H-CeO_2_@Ce6@PDA@GOx NPs under the same conditions. As shown in Figure S9B, the viability of T98G cells was found as 69.84% when H-CeO_2_@PDA NPs were exposed to NIR laser at 808 nm due to the temperature elevation triggered by photothermal response of H-CeO_2_@PDA NPs. However, the cell viability decreased to 42.03% when H-CeO_2_@Ce6@PDA@GOx NPs with the same concentration were exposed to NIR laser under the same conditions (Figure 8B). The enhancement of starvation function originated from higher glucose oxidation rate at higher temperatures obtained by the photothermal effect of STA is likely a reason for the increase of cell death. On the other hand, an increase in the ^·^OH radical generation rate is also expected depending on the increase in glucose oxidation rate induced by the same temperature elevation, which also increases the cell death by CDT.

(iii) Low cell viabilities between 10.6 and 13.1% were achieved using H-CeO_2_@Ce6@PDA@GOx NPs in the concentration range of 1–2 mg/mL, in the synergistic actions containing ST, PDT, CDT, and PTT carried out without using any chemotherapeutic agent.

(iv) With the low concentrations of H-CeO_2_@Ce6@PDA@GOx NPs in the range of 0.1–0.5 mg/mL, no significant difference is observed between the cell viabilities obtained with ST + CDT + PDT and ST + CDT + PTT functions (Figure 8B). However, the cell deaths obtained with ST + CDT + PTT were higher with respect to those of ST + CDT + PDT at higher concentrations of H-CeO_2_@Ce6@PDA@GOx NPs in the range of 1–2 mg/mL. This difference likely occurs due to enhancement of starvation and chemodynamic functions depending on higher temperature obtained with more appreciable photothermal effect originated from higher concentration of STA.

These findings show that H-CeO_2_@Ce6@PDA@GOx NPs is a new, effective STA which is capable of providing O_2_ evolution for alleviation of hypoxia and considerable *in vitro* T98G cell death by a combinatorial therapy including ST, PDT, PTT, and CDT without using any chemotherapeutic agent.

## 4 Conclusions

A new staged shape templating sol–gel method that can be initiated using uniform cross-linked polymethacrylate nanoparticles with cation exchanger functionality as the seed was developed for the synthesis of hollow, uniform, and mesoporous CeO_2_ NPs. This protocol is suitable for the production of different metal oxide nanoparticles such as silicon dioxide, manganese oxide, and copper oxide. The cation exchanger functionality allows for facile and effective adsorption of metal cations onto the seed, and the metal oxide shell can be formed on the seed surface by using an oxidation agent. The size and porous properties of hollow nanoparticles can be tuned by adjusting the size of the seed obtained by the precipitation copolymerization of methacrylate-based monomers and the formation conditions of metal oxide shell on seed nanoparticles. The synergistic therapy studies with some other metal oxide hollow nanoparticles produced by the synthetic protocol described in this work are still in progress.

The proposed construction of STA allowed us to observe an appreciable enhancement in the PDT function due to O_2_ evolution by the decomposition of glucose via the cascade enzyme system. Higher cell deaths were also obtained when ST+CDT functions of STA were used together with PTT with respect to PTT alone.

The effectiveness of synergistic therapy should also be higher in the presence of hypoxia relief when H-CeO_2_@Ce6@PDA@GOx NPs are loaded with a chemotherapeutic agent. H-CeO_2_@Ce6@PDA@GOx NPs have considerable pore volume and surface area originating from their mesoporous structure. The combination of O_2_ generation ability with satisfactorily high pore volume and high surface area is a superiority for H-CeO_2_@Ce6@PDA@GOx NPs loaded with a chemotherapeutic agent. In principle, a higher amount of antitumor agent can be loaded onto an STA with higher pore volume (also higher surface area). More effective chemotherapeutic function is expected when a higher amount of antitumor agent is released to the tumor microenvironment containing a higher concentration of O_2_ provided by the cascade enzyme system on the STA.

The hollow mesoporous character of synthesized STA also allows the loading of different agents, including chemotherapeutics, contrast agents, or targeting agents, etc. in addition to photosensitizers and enzymes. Hence, the synthesis of a theranostic agent with enhanced therapy performance can be potentially achieved by performing small structural modifications on the designed nanostructure containing mesoporous H-CeO_2_ and PDA compartments.

## Supporting Information Available

Materials; synthesis of H-CeO_2_ NPs; characterization of H-CeO_2_@Ce_6_@PDA@GOx NPs; catalase-like and peroxidase-like activity of H-CeO_2_@PDA NPs; glucose oxidase activity (GOx) of H-CeO_2_@Ce6@PDA@GOx NPs; photothermal properties of H-CeO_2_@Ce6@PDA@GOx NPs; photodynamic response of H-CeO_2_@Ce6@PDA@GOx NPs; combined photothermal, photodynamic, and starvation therapy performance of H-CeO_2_@Ce6@PDA@GOx NPs; morphological properties of H-CeO_2_ and H-CeO_2_@Ce6@PDA@GOx NPs (Table S1); EDX images of H-CeO_2_ and H-CeO_2_@Ce6@PDA@GOx NPs (Figure S1); X-ray photoelectron spectroscopy of H-CeO_2_ and H-CeO_2_@Ce6@PDA@GOx NPs (Figure S2) and (Table S2); GOx activity of H-CeO_2_@PDA@GOx NPs (Figure S3); catalase- and peroxidase-like activities of nanoparticles (Figures S4 and S5); determination of time constant for photothermal behavior of H-CeO_2_@Ce@PDA@GOx NPs (Figure S6); and controls for synergistic therapy experiments (Figures S7–S9) (PDF)
