# Supplementary material for: In Vitro Synergistic Photodynamic, Photothermal, Chemodynamic, and Starvation Therapy Performance of Chlorin e6 Immobilized, Polydopamine-Coated Hollow, Porous Ceria-Based, Hypoxia-Tolerant Nanozymes Carrying a Cascade System
Source: ACS Appl Bio Mater. 2024 Feb 21;7(5):2781–93. doi: 10.1021/acsabm.3c01181 (PMC11110068; doi:10.1021/acsabm.3c01181)
Supplement: Supplementary file 1 — mt3c01181_si_001.pdf [file mt3c01181_si_001.pdf]

Supporting Information

In-vitro synergistic photodynamic, photothermal, chemodynamic and starvation therapy performance of chlorin e6 immobilized, polydopamine coated hollow, porous ceria based, hypoxia tolerant nanozyme carrying a cascade system

Çağıl Zeynep Süngü Akdogan[1,2], Esin Akbay Çetin[3], Mehmet Ali Onur[1,3],

Selis Önel[1,4], Ali Tuncel[1,4,]*

1 Hacettepe University, Bioengineering Division, Ankara 06800, Turkey

2 Hacettepe University, Graduate School of Science and Engineering, Ankara 06800, Turkey

3 Hacettepe University, Department of Biology, Ankara 06800, Turkey

4 Hacettepe University, Chemical Engineering Department, Ankara 06800, Turkey

* Corresponding author. Email: atuncel@hacettepe.edu.tr

Number of pages: 17

Number of figures: 9

Number of tables: 2

S1. Materials

All the chemicals used for preparation of poly(glycerol dimethacrylate-co-methacrylic acid), poly(GDM-co-MA) particles and hollow CeO_2 nanoparticles (H-CeO_2 NPs) were purchased from Sigma-Aldrich, USA. Chlorin e6 is (Ce6) obtained from Medkoo, USA. Dopamine hydrochloride, tris(hydroxymethyl)aminomethane, trisodium citrate, o-phenylenediamine (OPDA), hydrogen peroxide (50 % w/w), hydrochloric acid (HCl, 37.5 % w/w), titanium (IV) chloride ($TiCl_4$), acetonitrile (ACN), ethanol and glucose oxidase (GOx, Catalog No: G6125, Type II, >15.000 units/g solid, *Aspergillus niger*) were purchased from Sigma-Aldrich. T98G human glioblastoma cells and L929 subcutaneous connective tissue cells) were supplied from American Type Culture Collection (ATCC, Manassas, VA, USA). High glucose Dulbecco's Modified Eagle's Medium (DMEM)/Ham's F12, 10 % (w/w) fetal bovine serum (FBS) and DMEM-F12 were purchased from Biochrom AG, Berlin, Germany. Phosphate buffered saline (PBS), dimethyl sulfoxide (DMSO), 0.25% trypsin–EDTA, acridine orange (AO), propidium iodide (PI), 3-(4,5-dimethylthiazol-2-yl)-2,5-diphenyl tetrazolium bromide (MTT), 1,3-diphenylisobenzofuran (DPBF), 2,7-dichlorofluorescein diacetate (DCF-DA) and penicillin/streptomycin (P/S) were supplied from Sigma-Aldrich. All synthesis runs and *in-vitro* experiments were conducted using deionized (DI) water with a resistivity of 18 MΩcm obtained from Direct-Q3, Millipore, U.S.A

S2. Synthesis of H-CeO_2 NPs

In this protocol, uniform-crosslinked poly(GDM-co-MA) NPs were used as the template. A precipitation polymerization was used for synthesis of poly(GDM-co-MA) NPs [1]. Typically, glycerol dimethacrylate (GDM) (1 mL) was copolymerized with methacrylic acid (MA) (0.27 mL) using AIBN (0.08 g) as the initiator in a continuous medium containing toluene (97.4 mL) and acetonitrile (ACN) (32.6 mL). The copolymerization was carried out at 70°C for 24 h in a

Pyrex® sealed glass reactor placed in a temperature-controlled shaking water bath. Poly(GDM-co-MA) NPs were washed with ethanol using a successive centrifugation-decantation protocol. Finally, Poly(GDM-co-MA) NPs were washed with and redispersed in deionized (DI) water.

For the synthesis of H-CeO_2 NPs, the continuous medium was prepared by dissolving $Ce(NO_3)_3 \cdot 6H_2O$ (600 mg) in an ethanol (30 mL)/water (28 mL) solution by ultrasonication. Poly(GDM-co-MA) NPs (400 mg) were dispersed in the solution and the dispersion was stirred at 300 rpm for 6 h. Hexamethylenetetramine (HMTA, 800 mg in 5.0 mL DI water) was injected and temperature of the reaction medium was elevated to 75 °C with a heating rate of 2°C/min. The reaction for the formation of CeO_2/polymethacrylate composite NPs was continued at 75°C for 8 h under magnetic stirring at 400 rpm. The composite NPs were recovered by centrifugation at 5000 rpm for 10 min and washed with ethanol and water by using a centrifugation/decantation protocol. After drying of composite NPs at 80°C for overnight, almost uniform-porous H-CeO_2 NPs were obtained by removing polymethacrylate template from the composite NPs by calcination at 550°C for 2 h with a heating rate of 2°C/min [2].

S3. Characterization of H-CeO_2@Ce6@PDA@GOx NPs

The porous properties (i.e. specific surface area, mean pore size and pore size distribution) were determined by nitrogen physisorption method using Brunauer-Emmett-Teller (BET) model in surface area and pore size analyzer (Quantachrome, Nova 2200E, UK). The surface structure and the mean size of H-CeO_2 and H-CeO_2@Ce6@PDA@GOx NPs were analyzed by Scanning Electron Microscope (SEM, Tescan, Czech Republic). The surface chemistry was analyzed by X-ray photoelectron spectroscopy (XPS, K-Alpha XPS system, Thermo Fischer Scientific, USA). The crystalline structure was evaluated by X-ray diffraction (XRD) spectrophotometer (Rigaku Ultima-IV, Japan).

S4. Catalase-like and peroxidase-like activity of H-CeO_2@PDA NPs

Catalase (CAT)-like activity of H-CeO_2 NPs or H-CeO_2@PDA NPs was determined by following the decomposition of H_2O_2 using a colorimetric protocol. Briefly, H-CeO_2 or H-CeO_2@PDA NPs (10 mg) were dispersed in an aqueous H_2O_2 solution (5.0 mL) prepared with an initial H_2O_2 concentration in the range of 0.05-1.0 mM. The reaction medium was rotated at 50 rpm for 30 min at room temperature, in the dark. The samples were periodically removed from the reaction medium and centrifuged at 5000 rpm for 5 min. H_2O_2 concentration in the supernatant obtained from the sample taken at a certain time was determined by measuring the absorbance of $TiCl_4/H_2O_2$ complex at 414 nm, in a UV-Vis spectrophotometer. The initial H_2O_2 consumption rate was calculated based on the variation of complex concentration with the time for first 1 min of the reaction period. Michaelis–Menten constants for catalase-like activity of H-CeO_2 or H-CeO_2@PDA NPs were determined by Lineweaver–Burk plots.

For the determination of peroxidase (POD)-like activity of H-CeO_2 NPs and H-CeO_2@PDA NPs, H-CeO_2 NPs or H-CeO_2@PDA NPs (2.0 mg/mL) were dispersed in OPDA solution (8.0 mL) prepared with Tris buffer (50 mM, pH 7.0) applying ultrasonication for 1 min. H_2O_2 solution (8 μL, 50 % w/w) was added to the dispersion. The reaction medium was rotated at 50 rpm for 30 min at room temperature (22°C). The samples were withdrawn from the reaction medium at prescribed times and centrifuged for the separation of H-CeO_2 NPs or H-CeO_2@PDA NPs from the liquid part. The absorbance of the supernatant was measured at 416 nm in a UV-Vis spectrophotometer (Thermoscientific, Genesys 150, USA). Initial OPDA consumption rates with different OPDA concentrations were calculated using the expression given in an earlier work [3,4]. OPDA solutions were prepared with the initial concentrations in the

range of 250–2000 μM and used for the determination of Michaelis-Menten model parameters for catalase-like activity of H-CeO_2 NPs or H-CeO_2@PDA NPs.

S5. Glucose oxidase activity (GOx) of H-CeO_2@PDA@GOx NPs

H-CeO_2@PDA@GOx NPs (12 mg) were washed and dispersed in citrate buffer (3.0 mL, 100 mM, pH 5) including glucose at different concentrations ranging between 5.5-55.5 mM. The resulting dispersion was shaken at a rate of 100 cpm, in the dark, for 1 h at 37°C. OPDA solution (2.0 mM, 3 mL) was prepared with citrate buffer at pH 5.0 and added into the dispersion. The reaction medium was shaken at 120 cpm for 30 min. The absorbance of the supernatant was measured at 416 nm in a UV-Vis spectrophotometer (Thermoscientific, Genesys 150, USA) by following the methodology used to determine the peroxidase-like activity. Initial glucose consumption rates were calculated based on the variation of OPDA concentration with the time for the initial course of reaction, obtained with the samples from the glucose oxidations performed with different initial glucose concentrations. Michaelis-Menten model parameters for GOx activity of H-CeO2@PDA@GOx NPs were calculated using Lineweaver-Burk plot [5,6].

S6. Photothermal properties of H-CeO_2@Ce6@PDA@GOx NPs

The dispersions were prepared at different H-CeO_2@Ce6@PDA@GOx NPs concentrations ranging between 0.1-2.0 mg/mL. Temperature of the aqueous dispersion was measured against time by means of a thermocouple fixed at the center of the container. After a temperature elevation period of 5 min, the NIR laser was turned off and the temperature of the aqueous dispersion was again recorded against time during the cooling process. The consecutive heating/cooling operations were repeated five times to assess the thermal stability of H-CeO_2@Ce6@PDA@GOx NPs.

Photothermal conversion efficiency (η) was calculated for H-CeO_2@Ce6@PDA@GOx NPs according to the time constant method, using the following equations [4,7].

$$\eta = \frac{hS\,(T_{max}-T_{Surr})-Q_{Dis}}{I(1-10^{-A_{808}})} \qquad \text{(Eqn. S1)}$$

$$\theta = \frac{T-T_{Surr}}{T_{max}-T_{Surr}} \qquad \text{(Eqn. S2)}$$

$$hS = \frac{m_D C_D}{\tau_s} \qquad \text{(Eqn. S3)}$$

T_{max} and T_{Surr} is the maximum temperature and the ambient temperature, respectively. S and h are the surface area of container and the heat transfer coefficient, respectively. A_{808} is absorbance at 808 nm. Q_{Dis} is heat input rate originated from the light-absorption of solvent determined in the absence of microspheres. τ_s is the time constant, which is determined by the linear regression of cooling curve, m_D is solution mass, C_D is specific heat.

S7. Photodynamic response of H-CeO_2@Ce6@PDA@GOx NPs

Briefly, H-CeO_2@Ce6@PDA@GOx NPs (5 mg/mL) were dispersed in DPBF solution (30 μg/mL in acetonitrile) in the dark [8]. The solution was irradiated with a red LED at 650 nm (0.8 Watt) as the visible light source. Absorption spectra of supernatants obtained by centrifuging the samples taken at various irradiation times (0, 1, 5, 10, and 20 min) were recorded in a UV−Vis spectrophotometer (Thermoscientific, Genesys 150, USA). In the control run, only DPBF solution alone, without including H-CeO_2@Ce6@PDA@GOx NPs was also irradiated with the same LED and the absorption spectra were recorded by the same method given above.

S8. Combined photothermal, photodynamic, chemodynamic and starvation therapy performance of H-CeO_2@Ce6@PDA@GOx NPs

Human glioblastoma cells (T98G) were cultured in a high glucose-DMEM medium containing 10 % (w/w) fetal bovine serum (FBS) and 1 % (w/w) penicillin/streptomycin (P/S) at 37 °C in

5% (v/v) CO_2 atmosphere. The culture media was refreshed every two days and subcultured before the synergistic therapy runs. Synergistic therapy experiments with H-CeO_2@PDA NPs, H-CeO_2@Ce6@PDA NPs and H-CeO_2@Ce6@PDA@GOx NPs were conducted in 96 well plates with a cell density of 2x10^4 cells/well [9]. Typically, H-CeO_2@Ce6@PDA@GOx NPs were incubated at different concentrations (0.1-2.0 mg/mL) with T98G cells in a fresh culture medium (200 µL, 2 % FBS). The medium was irradiated with a visible light source (red LED at 650 nm) for 7 min followed by NIR laser (808 nm) for 5 min. The fresh culture medium was applied and the cells were incubated at 37°C for 24 h. The cell viability was determined using the 3-(4,5- dimethylthia-zol-2-yl)-2,5-diphenyltetrazolium bromide (MTT) assay. The culture medium was replaced with the working medium (200 µL, 10 % w/w MTT), and the cells were incubated for 4 h in the dark. Isopropyl alcohol was replaced with the previous medium to dissolve the formazan crystals. Absorbance of the resulting solution (A) was measured at 570 nm on a microplate spectrophotometer (µQuantTM, BiotekW Instruments Inc, USA) [9-11]. The percentage of viable cells was calculated using Eq. (1).

$$Percentage\ of\ cell\ viability\ (\%) = \frac{A_{treated\ group}}{A_{control\ group}} x100 \qquad \text{(Eqn. S4)}$$

For following the cell death induced by synergistic therapy with dual cell staining using acridine orange/propidium iodide (AO/PI) system, 2x10^4 cells/well were cultured in 96 well plates. Following to consecutive application of LED and NIR laser for the periods given above, the fresh culture medium was applied and the cells were incubated for 24 h at 37°C. The medium was replaced with the dye solution (100 µL, 1:1, v/v) and incubated for 2 min. The cells were washed with PBS twice and examined using an inverted fluorescence microscope (Olympus IX70, Japan) in the dark. In order to see a possible cytotoxic effect, a control run was done using H-CeO_2 NPs without using LED and NIR laser under identical conditions [8-10].

For the determination of *in-vitro* cytotoxicity of H-CeO_2 NPs and H-CeO_2@Ce6@PDA@GOx NPs using L929 subcutaneous connective tissue cell line. L929 cells were cultured in DMEM-

F12 medium containing 10 % w/w FBS and 1% w/w P/S at 37°C under 5% (v/v) CO_2 atmosphere and then seeded into 96-well culture plates. L929 cells with a cell density of $2x10^4$ cells/well were interacted with H-CeO_2 NPs and H-CeO_2@Ce6@PDA@GOx NPs for 15 min, by varying the concentration of both types of NPs in the range of 0.1-2.0 mg/mL. The fresh culture medium was applied and L929 cells were then incubated at 37°C for 24 h. The viability of L929 cells was determined using MTT assay and live/dead cell images were obtained by dual cell staining with AO/PI system as also described above for T98G cells.

S9. Morphological properties of H-CeO_2 and H-CeO_2@Ce6@PDA@GOx NPs

Table S1. Size and porous properties of H-CeO_2 and H-CeO_2@Ce6@PDA@GOx NPs.

**Sample**	**D_p (nm)**	**SSA (m^2/g)**	**Pore volume (cm^3/g)**	**Mean pore diameter (nm)**
H-CeO_2	540	49.18	0.162	23.02
H-CeO_2@Ce6@PDA@GOx	550	34.42	0.133	16.07

D_p: Mean nanoparticle size, SSA: Specific surface area.

S10. EDX images of H-CeO_2 and H-CeO_2@Ce6@PDA@GOx NPs

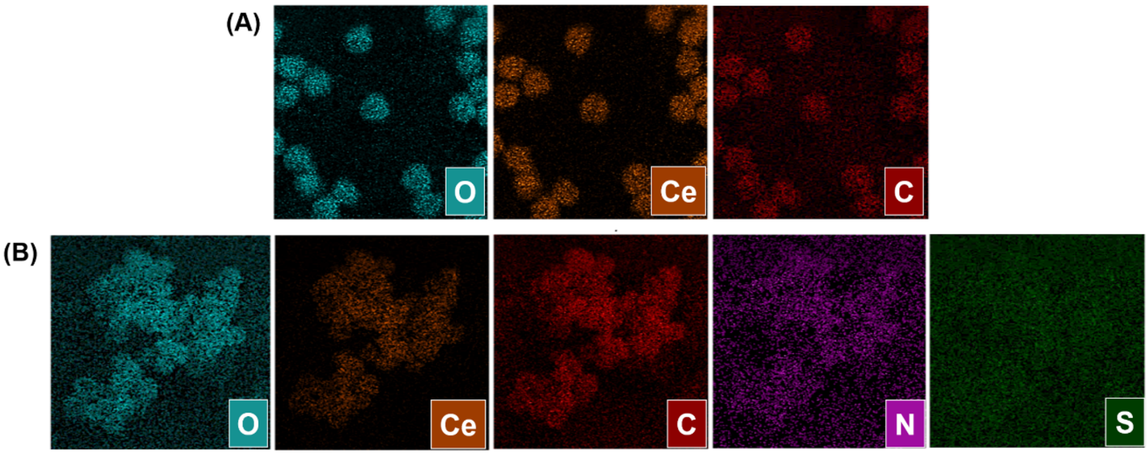

Figure S1. EDX images of (A) H-CeO_2 and (B) H-CeO_2@Ce6@PDA@GOx NPs.

S11. X-ray photoelectron spectroscopy of H-CeO_2 and H-CeO_2@Ce6@PDA@GOx NPs

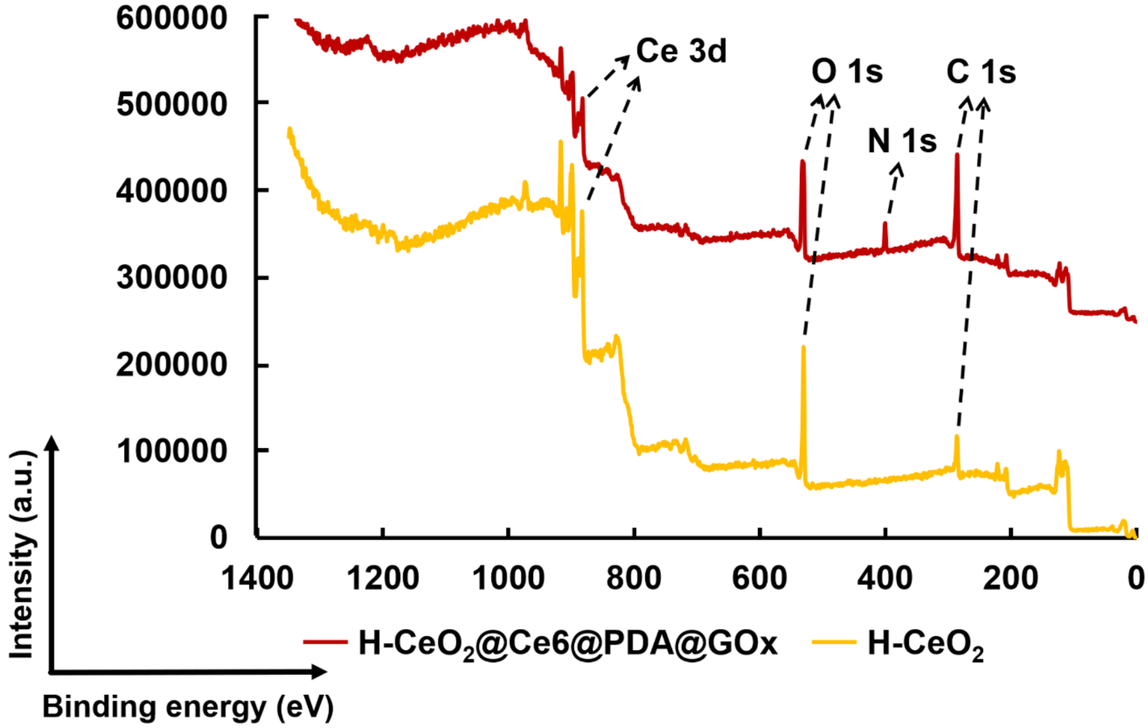

Figure S2. Survey XPS spectra of H-CeO_2 and H-CeO_2@Ce6@PDA@GOx NPs.

Table S2. Surface atomic compositions of bare H-CeO_2 and H-CeO_2@Ce6@PDA@GOx NPs determined by XPS.

	Surface atomic percentage (%)					
**Sample**	**Ce 3d (Total)**	**Ce 3d (Ce(III))**	**Ce 3d (Ce(IV))**	**C 1s**	**O 1s**	**N 1s**
H-CeO_2	16.47	3.46	13.01	34.63	48.89	-
H-CeO_2@Ce6@PDA@GOx	3.78	1.17	2.61	56.87	31.03	8.34

S12. GOx activity of H-CeO_2@PDA@GOx NPs

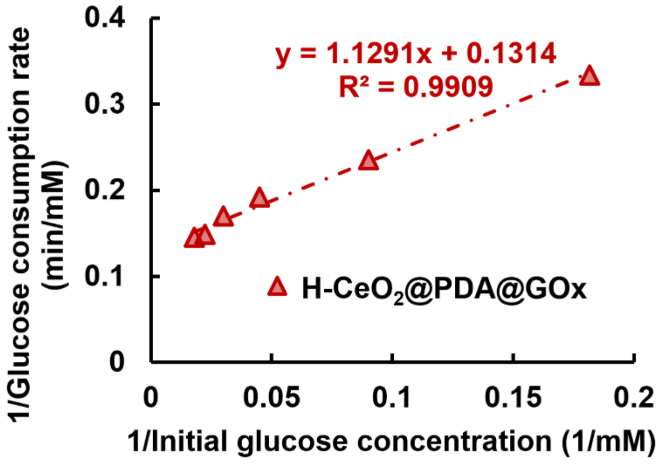

Figure S3. Lineweaver-Burk plot for GOx activity of H-CeO_2@PDA@GOx NPs. Nanozyme concentration: 2.0 mg/mL. Temperature: 37°C.

S13. Catalase- and peroxidase- like activities of nanoparticles

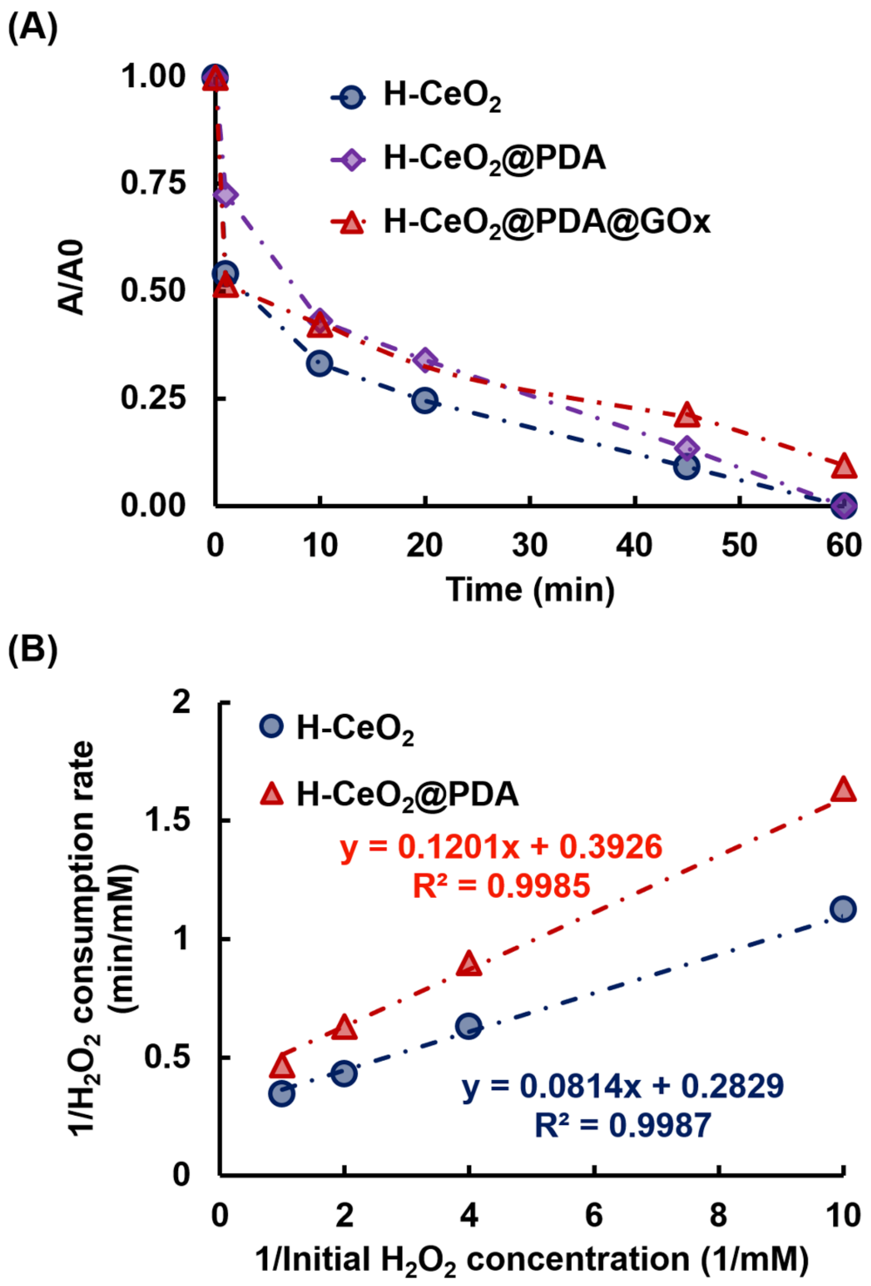

Figure S4. (A) H_2O_2 decomposition profiles obtained with H-CeO_2, H-CeO_2@PDA and H-CeO_2@PDA@GOx NPs. Nanozyme concentration: 2.0 mg/mL. Temperature: 22°C. (B) Lineweaver-Burk plot for catalase-like activity of H-CeO_2 and H-CeO_2@PDA NPs. Nanozyme concentration: 2.0 mg/mL. Temperature: 22°C

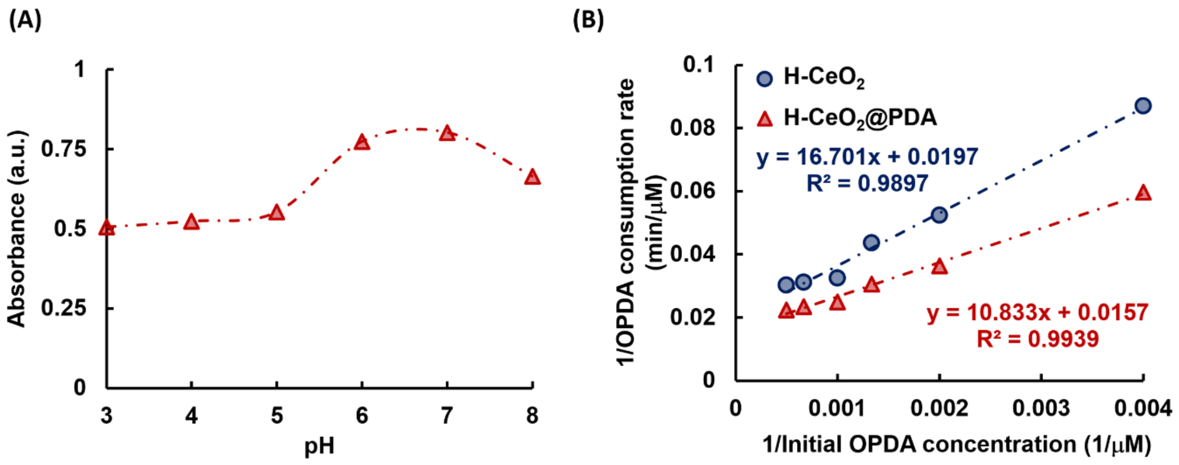

Figure S5. (A) Effect of pH on peroxidase-like activity of H-CeO_2@PDA NPs. OPDA concentration: 1000 μM, Nanozyme concentration: 2.0 mg/mL. Temperature: 22°C. (B) Lineweaver-Burk plot for peroxidase-like activity of H-CeO_2 NPs and H-CeO_2@PDA@PDA NPs. Nanozyme concentration: 2.0 mg/mL. Temperature: 22°C.

S14. Determination of time constant for photothermal behavior of H-CeO_2@Ce6@PDA@GOx NPs

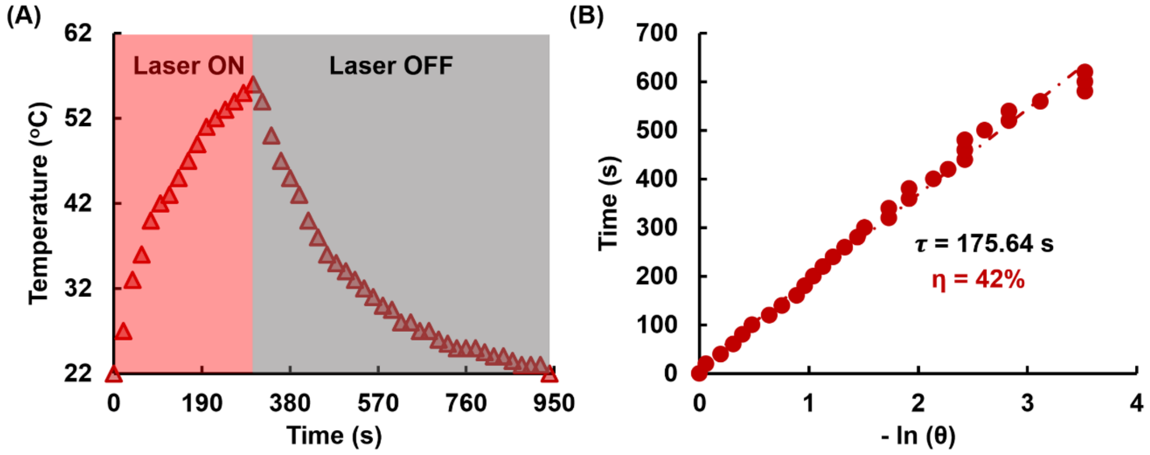

Figure S6. **(A)** Heating-cooling cycle obtained with H-CeO_2@Ce6@PDA@GOx NPs (2 mg/mL) under irradiation with NIR laser (808 nm). Heating and cooling curves are shown by

red and grey colors, respectively. **(B)** The time constant, τs obtained by linear fitting between -ln(θ) vs. time.

S15. Controls for synergistic therapy experiments

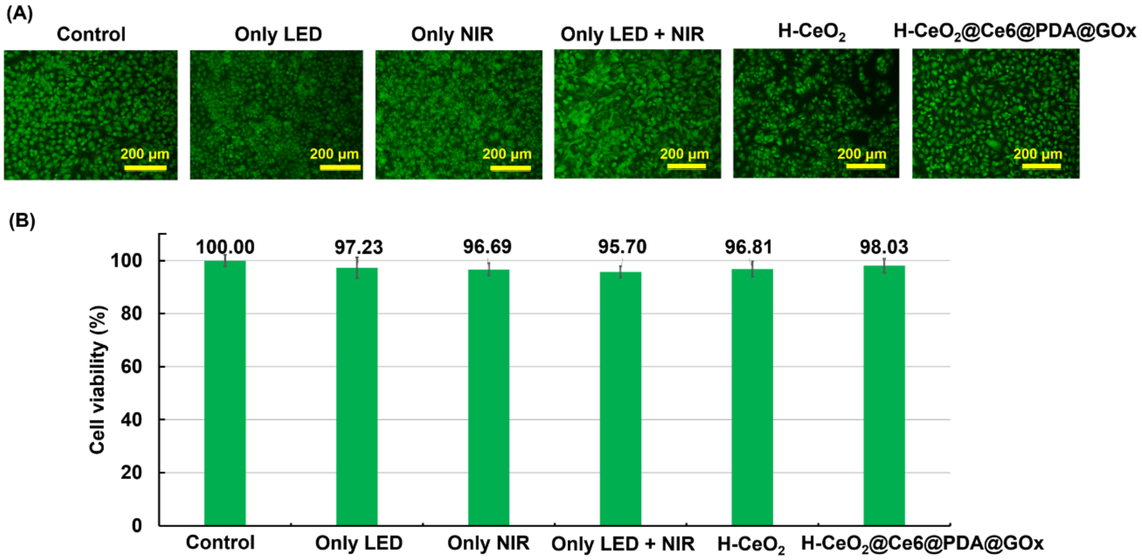

Figure S7. The viability of T98G cells against red LED (650 nm) and NIR laser (808 nm) irradiation. T98G concentration: $2x10^4$ cells/well, LED irradiation time: 7 min, NIR laser irradiation time: 5 min.

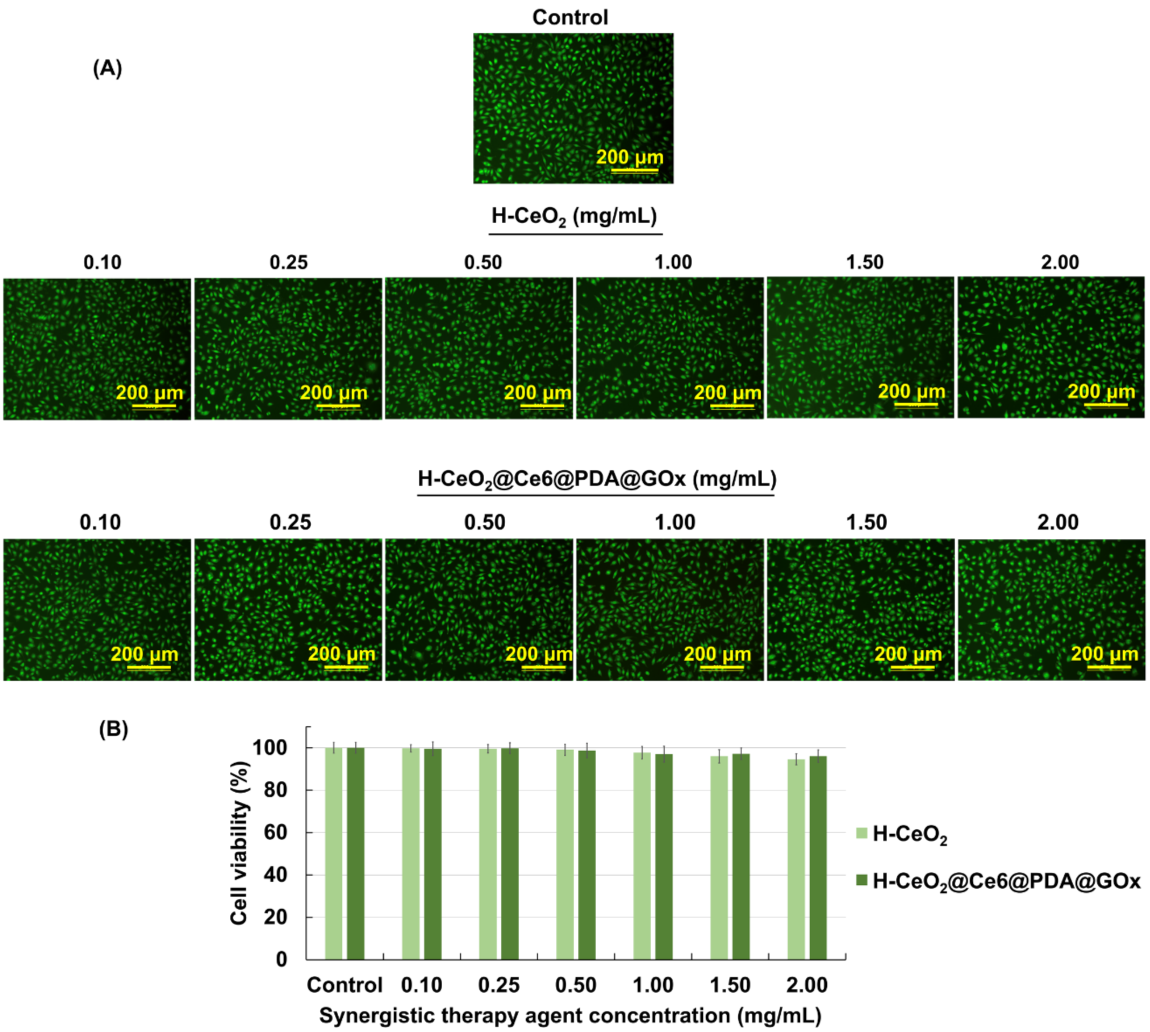

Figure S8. The viability of L929 cells in the presence of H-CeO_2 NPs and H-CeO_2@Ce6@PDA@GOx NPs with different concentrations. L929 cell density: $2x10^4$ cells/well. (A) Live/dead L929 cell images obtained by dual cell staining with AO/PI system, after treatment with H-CeO_2 and H-CeO_2@Ce6@PDA@GOx NPs at different concentrations. Scale bar: 200 μm. (B) MTT results demonstrating the viability of L929 cells after treatment with H-CeO_2 and H-CeO_2@Ce6@PDA@GOx NPs at different concentrations. The control image was taken in the absence of H-CeO_2 and H-CeO_2@Ce6@PDA@GOx NPs.

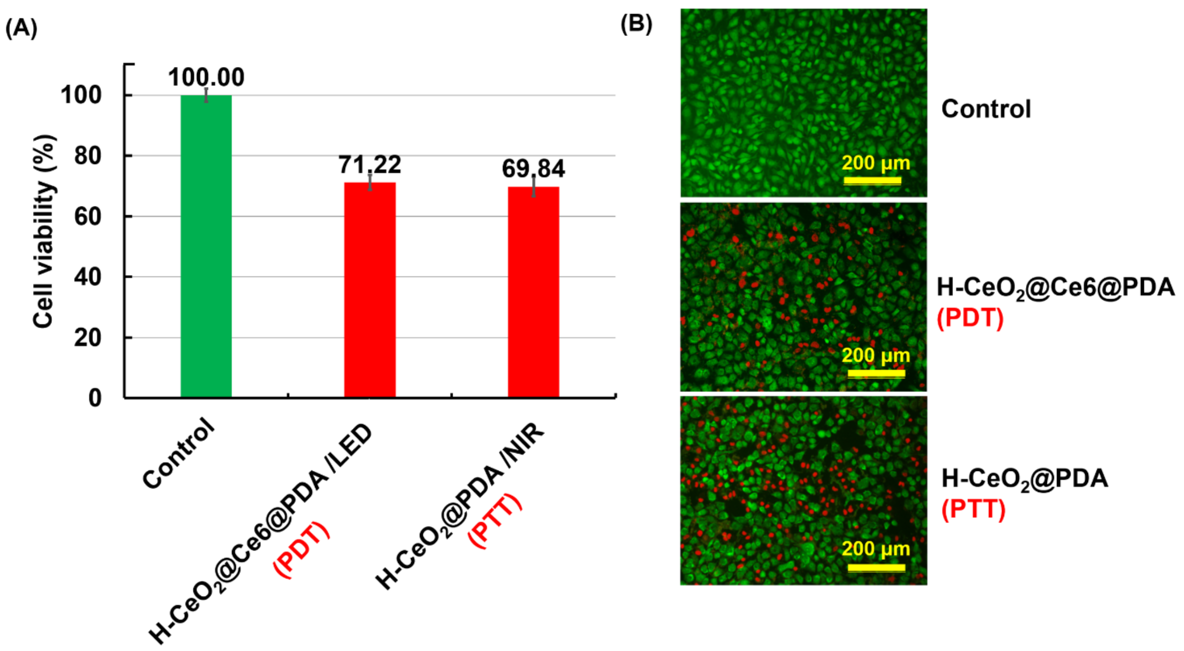

Figure S9. (A) Representative live/dead cell images of T98G cells stained with AO/PI after treatment with H-CeO_2@PDA NPs and H-CeO_2@Ce6@PDA NPs using LED light at 650 nm for 7 min and NIR laser at 808 nm for 5 min, respectively. Scale bar: 200 μm., T98G cell concentration: $2x10^4$ cells/well, NP Concentration: 1.0 mg/mL. (B) MTT results for demonstrating the individual contributions of PTT and PDT for T98G cells, using H-CeO_2@PDA NPs and H-CeO_2@Ce6@PDA NPs, respectively.

References

(1) Saraçoğlu, B.; Uğuzdoğan, E.; Gölgelioğlu, Ç.; Tuncel, A., Synthesis of Monodisperse Glycerol Dimethacrylate-Based Microgel Particles by Precipitation Polymerization. *Ind. Eng. Chem. Res.* **2009,** *48* (10), 4844-4851. https://doi.org/10.1021/ie801572w

(2) Yıldırım, D.; Gökçal, B.; Büber, E.; Kip, Ç.; Demir, M. C.; Tuncel, A., A new nanozyme with peroxidase-like activity for simultaneous phosphoprotein isolation and detection based on metal oxide affinity chromatography: Monodisperse-porous cerium oxide microspheres. *Chem. Eng. J.* **2021,** *403*, 126357. https://doi.org/10.1016/j.cej.2020.126357

(3) Özcan, S.; Süngü Akdoğan, Ç. Z.; Polat, M.; Kip, Ç.; Tuncel, A., A new multimodal magnetic nanozyme and a reusable peroxymonosulfate oxidation catalyst: Manganese oxide coated-monodisperse-porous and magnetic core-shell microspheres. *Chemosphere* **2023,** *341*, 140034. https://doi.org/10.1016/j.chemosphere.2023.140034

(4) Sungu Akdogan, C. Z.; Gokcal, B.; Polat, M.; Hamaloglu, K. O.; Kip, C.; Tuncel, A., Porous, Oxygen Vacancy Enhanced CeO_{2-x} Microspheres with Efficient Enzyme-Mimetic and Photothermal Properties. *ACS Sustain. Chem. Eng.* **2022,** *10* (29), 9492-9505. https://doi.org/10.1021/acssuschemeng.2c01981

(5) Jing, W.; Kong, F.; Tian, S.; Yu, M.; Li, Y.; Fan, L.; Li, X., Glucose oxidase decorated fluorescent metal–organic frameworks as biomimetic cascade nanozymes for glucose detection through the inner filter effect. *Analyst* **2021,** *146* (13), 4188-4194. https://doi.org/10.1039/D1AN00847A

(6) Gökçal, B.; Kip, Ç.; Tuncel, A., One-pot, direct glucose detection in human whole blood without using a dilution factor by a magnetic nanozyme with dual enzymatic activity. *J. Alloys Compd.* **2020,** *843*, 156012. https://doi.org/10.1016/j.jallcom.2020.156012

(7) Liu, X.; Li, B.; Fu, F.; Xu, K.; Zou, R.; Wang, Q.; Zhang, B.; Chen, Z.; Hu, J., Facile synthesis of biocompatible cysteine-coated CuS nanoparticles with high photothermal conversion efficiency for cancer therapy. *Dalton Transactions* **2014,** *43* (30), 11709-11715.https://doi.org/10.1039/C4DT00424H

(8) Wang, J.; Yang, Y.; Xu, Y.; Zhao, L.; Wang, L.; Yin, Z.; Li, H.; Tan, H.; Liu, K., A dual enhanced anti-bacterial strategy based on high chlorin e6-loaded polyethyleneimine functionalized graphene. *RSC Adv.* **2021,** *11* (2), 739-744. https://doi.org/10.1039/D0RA07976F

(9) Keskin, S.; Çetin, E., Lavender Volatile Oil: A New Solvent for Propolis Extraction, Chemical Composition, Antioxidant Activity and Cytotoxicity on T98G Glioblastoma Cell Line. *J. Essent. Oil-Bear. Plants* **2020,** *23* (3), 514-521. https://doi.org/10.1080/0972060X.2020.1801518

(10) Murugan, C.; Park, S., Cerium ferrite@molybdenum disulfide nanozyme for intracellular ROS generation and photothermal-based cancer therapy. *J. Photochem. Photobiol. A: Chem.* **2023,** *437*, 114466. https://doi.org/10.1016/j.jphotochem.2022.114466

(11) Erfen, Ş.; Akbay Çetin, E., Therapeutic and Preventive Effects of Piperine and its Combination with Curcumin as a Bioenhancer Against Aluminum-Induced Damage in the Astrocyte Cells. *Neurotox. Res.* **2022,** *40* (6), 2027-2045. https://doi.org/10.1007/s12640-022-00600-9
